# The role of epigenetics in women’s reproductive health: the impact of environmental factors

**DOI:** 10.3389/fendo.2024.1399757

**Published:** 2024-09-13

**Authors:** Xinru Yu, Jiawei Xu, Bihan Song, Runhe Zhu, Jiaxin Liu, Yi Fan Liu, Ying Jie Ma

**Affiliations:** ^1^ College Of Pharmacy, Shandong University of Traditional Chinese Medicine, Jinan, Shandong, China; ^2^ College Of Traditional Chinese Medicine, Shandong University of Traditional Chinese Medicine School, Jinan, Shandong, China; ^3^ Medical College, Shandong University of Traditional Chinese Medicine, Jinan, Shandong, China; ^4^ The First Clinical College, Shandong University of Traditional Chinese Medicine, Jinan, Shandong, China

**Keywords:** epigenetics, women, reproductive, environmental factors, reproductive health

## Abstract

This paper explores the significant role of epigenetics in women’s reproductive health, focusing on the impact of environmental factors. It highlights the crucial link between epigenetic modifications—such as DNA methylation and histones post-translational modifications—and reproductive health issues, including infertility and pregnancy complications. The paper reviews the influence of pollutants like PM2.5, heavy metals, and endocrine disruptors on gene expression through epigenetic mechanisms, emphasizing the need for understanding how dietary, lifestyle choices, and exposure to chemicals affect gene expression and reproductive health. Future research directions include deeper investigation into epigenetics in female reproductive health and leveraging gene editing to mitigate epigenetic changes for improving IVF success rates and managing reproductive disorders.

## 1 Introduction

The World Health Organization (WHO) identifies Sexual and Reproductive Health (SRH) as integral to achieving the highest standard of health. Women’s reproductive health remains a significant global concern, impacting not only physical well-being but also societal development, economic growth, and public health. Women’s unique physiological structures and responses, including the glandular folds of their internal and external genitalia in a moist environment, create conditions conducive to pathogen survival. The presence of androgens in the body may promote the growth and proliferation of pathogens, significantly increasing the risk of tumor development (1). During menstruation, the disruption of the endometrial lining and the increased susceptibility to various infections during sexual intercourse further exacerbate women’s reproductive health challenges (2). According to WHO data, 40% of Chinese women suffer from various degrees of reproductive tract infections, with the prevalence among married women reaching up to 70%. This indicates that approximately 300 million women in China face reproductive health issues, a figure significantly higher than the incidence rate of common colds. The repercussions of reproductive health issues on women’s lives and careers are profound, causing immense distress and suffering. Common gynecological conditions include endometriosis, uterine fibroids, ovarian cysts, vaginitis, cervicitis, cervical erosion, pelvic inflammatory disease, adnexitis, functional uterine bleeding, breast diseases, infertility, and menstrual disorders. Notably, endometriosis affects 10% (190 million) of women of reproductive age worldwide (3). The majority of gynecological diseases, along with psychological factors such as work stress and environmental conditions, can adversely affect ovarian function. This leads to metabolic disorders, endocrine disruptions, and imbalances in estrogen and progesterone levels, triggering conditions such as melasma, wrinkles, constipation, acne, obesity, hyperlipidemia, and even carcinogenesis, accompanied by mental lethargy (4, 5). Hence, reproductive system issues directly impact human health.

Over the past decade, notable progress has been made in the prevention and treatment strategies for common diseases of the female reproductive system. However, current research on the impact of environmental factors through epigenetic mediation on women’s reproductive health remains fragmented. In this review, we summarize the latest findings on the influence of environmental factors on epigenetics and, consequently, on women’s reproductive health. This includes an overview of several common types of epigenetics and the potential cellular and molecular mechanisms involved. Furthermore, we discuss the impact of both internal and external environmental factors on the female reproductive system. Therefore, our aim is to gain a more comprehensive understanding of the pathophysiological processes and potential mechanisms related to female infertility diseases, with the goal of enhancing fertility and pregnancy outcomes in women of childbearing age.

## 2 An introduction to epigenetics

Epigenetics refers to the transmission of genetic information that does not involve changes to the DNA sequence. It operates through chemical modifications on the genome, such as histones post-translational modifications, DNA methylation, and hydroxymethylation, thereby altering the way genes are expressed. The mechanism by which DNA methylation leads to gene silencing is not yet fully understood; however, three main forms are commonly considered. First, DNA methylation affects the transcriptional activity of genes. It can directly hinder the recognition and binding of transcription factors to specific DNA sequences, thus inhibiting gene transcription (6). Second, the mechanism by which methylation leads to gene silencing involves the methylation of CpG islands located in promoters or other regulatory regions playing a role in gene repression. Methylation of CpG islands in non-coding promoter regions recruits sequence-specific methylated DNA binding proteins and histone deacetylases (HDACs), forming complexes that suppress transcription by obstructing the binding of transcription factors to their target sequences, thereby affecting transcription (7, 8). Finally, DNA methylation can alter chromatin structure to suppress gene expression, where highly methylated promoters cause chromatin to condense, further affecting transcription (9).

Epigenetic modifications can regulate the splicing and expression patterns of genes (10). For instance, numerous studies have shown that histones post-translational modifications can regulate the binding and activity of splicing factors, thus influencing gene splicing. Results obtained from real-time quantitative Polymerase Chain Reaction(PCR) analysis of 16 epigenetic remodeling markers in the epidermal cells of 14 patients after *in vitro* amplification compared to freshly isolated epidermal cells (ISO), indicated a significant reduction in the transcription levels of genes involved in DNA methylation and histones post-translational modifications in cells cultured to the second generation of keratinocyte formation.

Beyond the aforementioned effects, epigenetic modifications can also regulate genomic stability and genetic memory, enabling cells to stably express specific phenotypes (11). Further research and understanding of the mechanisms of epigenetics are crucial for unraveling important processes in organism development, environmental adaptation, and disease onset.

## 3 The potential impact of environmental factors on women’s reproductive health

Infertility is commonly defined as “a disease of the male or female reproductive system characterized by the failure to achieve a successful pregnancy after more than one year of regular, unprotected sexual intercourse.” External harmful environmental factors can impair women’s fertility. Non-gaseous pollutants, such as PM2.5, have a certain impact on female reproduction(12, 13). Gaseous pollutants (such as ozone (O_3_), sulfur dioxide (SO_2_), and nitrogen oxides (NOx)) can affect the endocrine system of women of childbearing age, leading to infertility and pregnancy complications, including reduced ovarian reserve (14, 15), uterine fibroids (16), and preeclampsia (17, 18).

Harmful chemicals also impact women’s reproductive health (19). Polycyclic aromatic hydrocarbons have been shown to interact with estrogen receptors, activating the aryl hydrocarbon receptor leading to changes in steroid functionality and anti-estrogenic activity, resulting in adverse pregnancy outcomes such as preterm birth, miscarriage, and embryonic developmental arrest (20).Studies indicate that the widespread use of the heavy metal cadmium can cause endocrine disruption in women, potentially directly affecting the development of oocytes, the development of the uterus, and ovarian function, leading to decreased fertility, spontaneous miscarriage, and other reproductive issues (21).

## 4 Defining the purpose and scope of a literature review

In recent years, a growing body of evidence suggests that environmental exposures can leave epigenetic marks on genes, with various environmental factors proven to induce global or specific epigenetic changes (266). In this review, we discuss the interactions between environmental factors related to women’s reproductive health risks and epigenetics. This includes more common epigenetic processes such as DNA methylation, histones post-translational modifications, and non-coding RNA. Key roles in genetic regulation are played by external environmental factors like heavy metals (cadmium), polycyclic aromatic hydrocarbons, air pollutants, and internal factors such as dietary and nutritional elements, as well as the influence of parental care and climate factors on epigenetics. Moreover, we comment on the regulation of female reproductive functions through epigenetics, such as the pathogenesis and regulatory processes related to Polycystic Ovary Syndrome (PCOS), Premature Ovarian Insufficiency (POI), and endometriosis mediated by epigenetics, elucidating the function of epigenetics as a mediator, bridging environmental factors and female reproduction. Besides the adverse effects on female reproductive functions, epigenetic processes and abnormal epigenetic marks can also impact offspring health. Therefore, understanding how maternal environmental factors can affect offspring health through epigenetic mechanisms is crucial for preventing and managing environmentally related women’s reproductive health issues and accelerating the application of epigenetics in reproductive medicine.

## 5 The fundamentals of epigenetics

Epigenetics, also referred to as character genetics, exogenetics, paragenetics, postgenetics, or topogenetics, such as DNA methylation and histone modifications. DNA methylation is termed a carrier of epigenetic information, whereas variations and modifications of histones can directly or indirectly impact the structure of local chromatin. These chemical modifications to chromatin are both inheritable and reversible.

### 5.1 Epigenetic mechanisms

#### 5.1.1 DNA methylation

DNA methylation is the most prevalent epigenetic regulatory mechanism, where methyl groups are covalently bonded to cytosine residues in DNA sequences through enzyme-mediated reactions catalyzed by specific methyltransferases. In vertebrates, three methylation states of DNA are recognized: a persistent hypomethylation state (22), an induced demethylation state, and a hypermethylation state, which is notably observed in the methylation modifications of the inactivated X chromosome (23). Recent studies have demonstrated that genome-wide hypermethylation can impede the epithelial-to-mesenchymal transition, thereby further inhibiting the healing of chronic wounds (24).

In mammals, DNA methylation involves the covalent transfer of a methyl group to the C-5 position of the cytosine ring in CpG dinucleotides. As a form of chemical modification, DNA methylation alters the structure of the cytosine residues, creating the “fifth base” - 5-methylcytosine (5mC), which is the most significant form of DNA methylation in mammals. DNA methylation is essential for the organism’s normal growth and development, including the formation of genetic imprints(11

) and the promotion of dispersed chromatin to become condensed. DNA methylation also impacts the normal expression of genes (25), leading to a decrease in gene transcriptional activity. The mechanism by which DNA methylation impedes gene transcription is complex: it interferes with the binding of transcription factors to promoters, thus blocking transcription (1). Moreover, transcription factors can recognize methylated DNA and reactivate gene transcription (26). DNA methylation is catalyzed by DNA methyltransferases (DNMTs), with DNMT1 and DNMT3 being the active enzymes responsible for establishing and maintaining DNA methylation (27). DNA methyltransferases add methyl groups to CpG islands using S-adenosyl methionine (SAM) as a substrate. Zhu Bing was the first to use the methylation profile of mouse oocytes to confirm that DNMT1 indeed functions as an initiating DNA methyltransferase (28).

*De novo* methylation refers to the addition of methyl groups to previously unmethylated cytosines under the action of DNMT3 methyltransferases. DNMT3A and DNMT3B are the primary enzymes for mammalian DNA methylation, known as *de novo* methyltransferases (29). DNMT3A-mediated DNA methylation plays an indispensable role in the spermatogenesis of male germ cells (30),Mutations in DNMT3A render it insensitive to the inhibition of H3K4me3, resulting in the aberrant methylation of promoter subgroups marked by H3K4me3 in mouse embryonic stem cells (ESCs). This aberrant methylation leads to the downregulation of associated genes (31).While the ectopic expression of DNMT3B can enhance the genome-wide methylation level of haploid embryonic stem cells, shorten the transition from the G2 phase to the M phase of cell mitosis, alleviate spontaneous diploidization of haploid cells, and extend the survival time of semiclone mice (32).Weinberg et al. (33) demonstrated that H3K36me2 is essential for the recruitment and maintenance of DNA methylation in intergenic regions by DNMT3A. In contrast, Shirane et al. (34) showed that H3K36me2, deposited by NSD1, plays a crucial role in *de novo* methylation in germ cells. Typically, *de novo* methyltransferases preferentially bind to CpG-rich regions that are not protected by H3K4 methylation. These enzymes with methyltransferase activity prioritize the methylation of unmethylated CpG dinucleotides. For instance, the flexible N-terminal guides DNMT3AA1 to its bivalent target catalytic methyltransferase domain, thereby regulating DNA methylation and gene expression (35). DNMT3L, while having no catalytic activity, can interact with DNMT3A and DNMT3B to regulate their activity (36) and is expressed only in specific rodents like mice, rats, gerbils, and hamsters (37). In human oocytes, it is transcriptionally silent and involved in the regulation of repetitive elements and imprinting in germ cells. Although DNMT3L cannot bind the methyl donor S-adenosyl-L-methionine (SAM), it facilitates the interaction of SAM with DNMT3A2, aiding the *de novo* methyltransferases (38).

DNMT1 is the key enzyme for maintaining DNA methylation (39), preserving the methylation pattern from the parental DNA strand to the daughter strand through cell division and DNA replication (40). The C-terminal domain of DNMT1 consists of two subdomains: the Target Recognition Domain (TRD), which identifies hemimethylated cytosines, and the methyltransferase domain.

DNMT1 preferentially methylates hemimethylated DNA, a process primarily facilitated by the TRD domain recognizing cytosines within hemimethylated DNA. Once the DNA methylation pattern is established, the DNA methyltransferase DNMT1 maintains this pattern during DNA replication. The faithful replication of the DNA methylation pattern during cell division makes it an ideal mechanism for preserving epigenetic memory.

A recent review on DNA methylation proposed a regulatory model for DNMTs, where *de novo* methyltransferases adopt an autoinhibitory conformation until they are locally activated upon binding to the N-terminal tail of histone H3. Additional research indicates that this regulatory principle is applicable not only to classical *de novo* methyltransferases but also to DNMT1 (41). In this context, the replication foci targeting sequence (RFT) domain interacts with conformational activators, such as UHRF1, thereby exposing the catalytic site (42, 43).

DNA methylation can suppress the activity of certain genes, while DNA demethylation induces the reactivation and expression of genes (267). TET enzymes play a key role in the DNA demethylation process (44), including TET1, TET2, and TET3. These enzymes convert 5-methylcytosine (5mC) into 5-hydroxymethylcytosine (5hmC) (45), which can be further oxidized to 5-formylcytosine (5fC) and 5-carboxylcytosine (5caC). Subsequently, the TET and base excision repair (BER) pathways intervene in the repair process, converting these modified bases back to unmethylated cytosine, thereby achieving DNA demethylation. Although the BER pathway is commonly considered the final step in DNA demethylation, there remains controversy regarding the specific enzymes and chemical intermediates formed during this process (46). The implications of DNA demethylation are increasingly recognized; for instance, aberrant DNA hypermethylation has been detected in the leptotene spermatocytes of some patients with non-obstructive azoospermia, suggesting that DNA demethylation can influence male meiotic recombination and fertility (47).

#### 5.1.2 Histone post-translational modification

**Figure 1:**
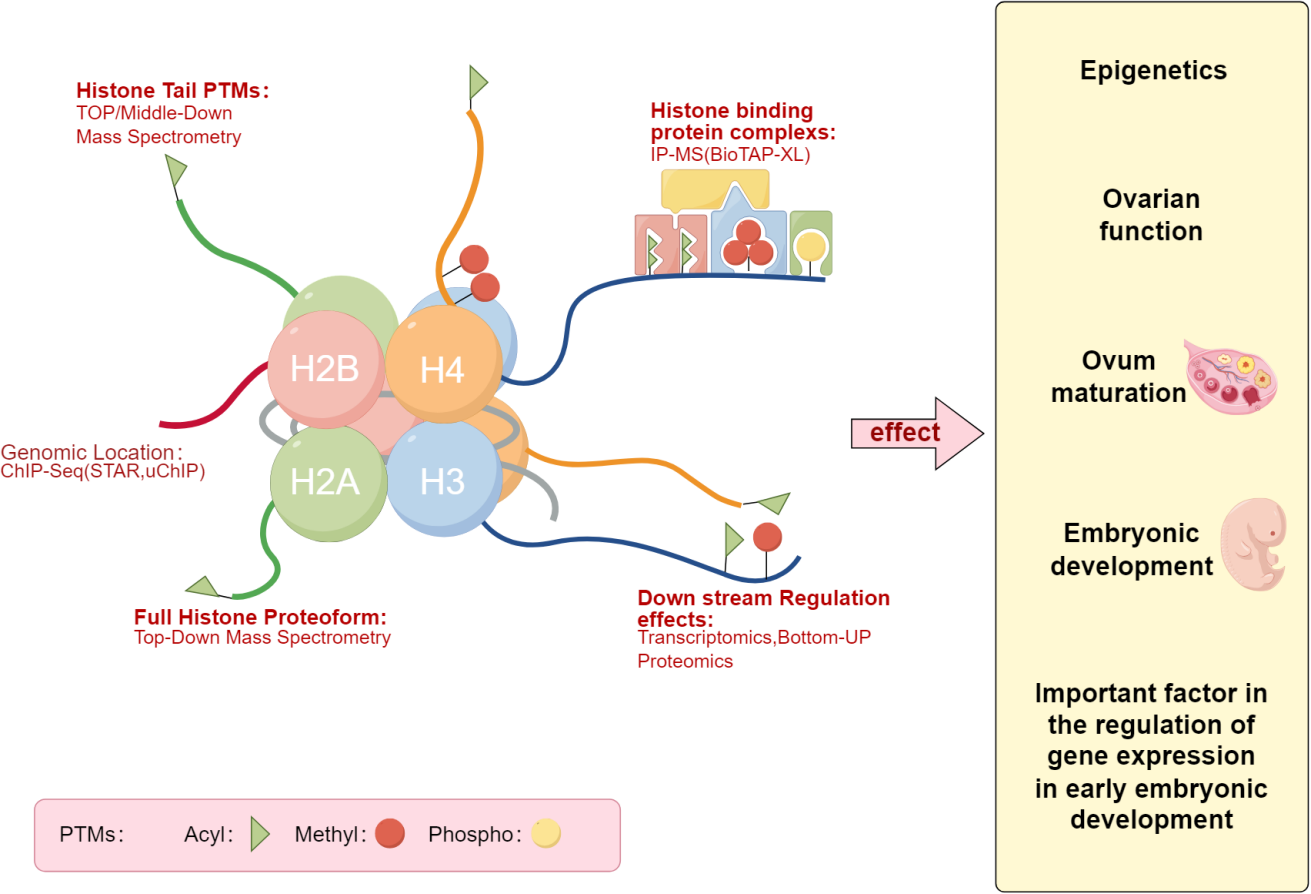
Histones post-translational modifications Map. The left panel describes the patterns of histones post-translational modifications, while the right panel lists the potential impacts of histones post-translational modifications on the pregnancy process, such as embryonic development (Draw by figdraw.).

Histones are primarily composed of a globular domain (48) and tails that protrude outside the nucleosome, made up of basic amino acids forming a fundamental structural protein. Two H2A-H2B dimers and one H3-H4 tetramer assemble into a histone octamer. Histones and DNA are the basic components of the nucleosome, providing appropriate sites for DNA winding when nuclear DNA is in a highly condensed state. Timothy J. Richmond and others determined the crystal structure of the chromatin nucleosome core particle, with a 2.8A high-resolution X-ray structure detailing the internal binding mode of the histone octamer and the superhelical organization of its surrounding 146 base pairs of DNA (49). Unmethylated nucleosome DNA spontaneously extends, forming four superhelical turns (50), a phenomenon that strongly evidences the crucial role of histone tails in maintaining the overall nucleosome structure (51).As illustrated in **Figure 1**, the diagram represents the spectrum of histones post-translational modifications. histones post-translational modifications mainly include methylation, acetylation, ubiquitination, sumoylation, and citrullination.

Methylation of histones that wrap DNA in eukaryotic chromosomes can have significant implications for human health. It occurs on all basic residues, including arginine (52), lysine (53), and histidine (54). Protein methylation can occur at the N-, O-, and S-centers of amino acid residues, with arginine and lysine residues being the most common sites of histone methylation. The effects of methylation on gene activity vary (55), depending on the modified amino residue (56), the genetic context of methylation, its level and pattern. Amino acid methylation primarily changes chromatin structure by increasing or decreasing DNA-histone interactions, leading to the activation or suppression of gene transcription. The core histone H3 plays a key role in nucleosome structure and gene expression regulation, being a primary site of histone methylation, along with other core histones like H2A, H2B, and H4, which are central components of the nucleosome and participate in histone methylation (57). During histone methylation, S-adenosylmethionine (SAM) (58) serves as the substrate, and under the catalysis of histone methyltransferases (HMTs, also known as “writers”), it transfers its methyl group to the lysine residues of histones (59). On the ϵ-amino group of lysine, it can be monomethylated, dimethylated, or trimethylated (60–62), referred to as me1, me2, and me3 (63). Currently, extensive research has been conducted on methylated histones, including H3K4, H3K9, H3K27, H3K36, H3K79, and H4K20 (64). Studies show that H3K4, H3K36, and H3K79 are mainly found in transcriptionally active regions of chromatin, playing a role in activating gene transcription. Conversely, H3K9, H3K27, and H4K20 often act as markers for gene transcription repression, closely associated with gene silencing (65–68).

Histone acetylation is a reversible process where acetyl groups are transferred by histone acetyltransferases (HATs), with lysine acetylation being a common form of histone acetylation. Simply put, this process involves adding an acetyl group to the positively charged lysine residues. Histone acetylation decreases the electrostatic affinity between histones and negatively charged DNA, loosening chromatin structure and activating open chromatin regions (69), making it easier for transcription factors and RNA polymerase to access DNA, thereby promoting transcription (70). Histone 1 is the most commonly acetylated protein, and H2A, H2B, H3, and H4 form the core of the nucleosome, referred to as core histones. Acetylation modifications within these core histones affect the dynamics of the nucleosome core particle (71), with the acetylation of H2B, H3, and H4 having minimal impact on the dynamics of the nucleosome core particle. In contrast, H2A shows significantly increased dynamics after acetylation modification (72). Histone acetylation primarily occurs at the amino-terminal lysine sites of H3 and H4, including H3K9ac, H3K14ac, H3K18ac, H3K23ac, H3K27ac, H3K56ac, H4K5ac, H4K8ac, H4K16ac, and H4K20ac (73), catalyzed by HATs such as CBP/p300, MOF, HBO1, or KAT6A (74). Previous extensive research has revealed a link between histone acetylation levels and the expression of pro-inflammatory cytokines and other antimicrobial products (75), with acetylases playing an indispensable role.

Acetyl-CoA is the donor of the acetyl group, produced by metabolic enzymes in the nucleus and having a direct effect on histone acetylation (76, 77). To date, studies have shown that three enzymes are crucial for maintaining acetyl-CoA levels: the Acyl-CoA Synthetase Short-Chain Family Member 2 (ACSS2) (78–80). Extensive research indicates that ACSS2 catalyzes the synthesis of acetyl-CoA from acetate, recruited to neuronal sites associated with organismal memory, crucial for memory consolidation (81). Furthermore, ACSS2 is an enzyme required for alcohol-induced neuron-specific gene expression and alcohol-associated associative learning (82). In summary, extensive research highlights the key role of ACSS2 in enhancing spatial memory and regulating histone acetylation.

Zhang et al. (83) were the first to discover histone lactylation, where lactate-derived histone lysine lactylation emerged as a novel epigenetic modification and demonstrated that histone lactylation directly stimulates chromatin gene transcription. Wan et al. reported the formation of cyclic immonium ions of lactylated lysine during tandem mass spectrometry analysis, enabling the identification of protein lactylation. Their findings highlighted that lactylation is common on glycolytic enzymes and is conserved on Aldolase A. Additionally, widespread lactylation was identified on Reductase SDR Family Member 7(DHRS7) in a draft of the human tissue proteome (84).

#### 5.1.3 Non-coding RNA

In eukaryotes, approximately 90% of genes are transcribed, of which only 1%-2% are responsible for encoding proteins, with the majority being transcribed into non-coding RNAs (ncRNAs). ncRNAs refer to RNA molecules transcribed from genes that do not have the capacity to encode proteins.

ncRNAs can be primarily classified into two forms: small ncRNAs (SncRNAs, 18-200 bp) and long ncRNAs (lncRNAs, >200 bp), neither of which can be translated into proteins. SncRNAs include small nucleolar RNAs (snoRNAs), PIWI-interacting RNAs (piRNAs), small interfering RNAs (siRNAs), microRNAs (miRNAs), circular RNAs (circRNAs), and extracellular RNAs (exRNAs) (85, 86). Extensive research on ncRNA biology has demonstrated that ncRNAs play a critical regulatory role in shaping cellular activity. ncRNAs include molecules that act as oncogenes or tumor suppressors, and their aberrant expression has been linked to carcinogenesis and metastasis regulated by epigenetic mechanisms (87–89). ncRNAs can regulate the transcription of individual genes or entire transcriptional programs, affecting the expression of hundreds to thousands of genes (90–92).

MicroRNA (miRNA) is one of the most extensively studied ncRNAs, consisting of single-stranded molecules approximately 20-24 bp in length. Thousands of miRNAs can function as tumor suppressors or oncogenes by base-pairing with complementary sequences in the 3’UTR of target mRNAs, inhibiting gene translation and leading to gene silencing (93). Competitive endogenous RNAs (ceRNAs) are common post-transcriptional regulators. Notably, long non-coding RNAs (lncRNAs) and circular RNAs (circRNAs) within the ceRNA category can induce gene silencing, affecting gene expression and thereby influencing tumor progression (94). lncRNAs regulate various physiological processes such as tumor cell invasion, migration, proliferation, and tumor microenvironment (TME) remodeling by modulating mRNA processes and gene transcription (95, 96). The unique circular structure of circRNAs, resulting from the back-splicing of pre-mRNA, confers them with exceptional stability. CircRNAs, as endogenous ncRNAs with their linear transcriptional 3’ and 5’ ends removed, are implicated in biological processes related to tumor suppression and carcinogenesis (97, 98).

### 5.2 Advances in research methods and techniques in epigenetics

Current research in epigenetics primarily focuses on three aspects: DNA methylation, histones post-translational modifications, and non-coding RNA. The methodologies for studying DNA methylation (99) are categorized into two main types. The first type examines the overall level of DNA methylation, including techniques such as whole-genome bisulfite sequencing (WGBs), methylation 450K array, and immunoprecipitation techniques (MeDIP). The second type investigates DNA methylation at specific sites; after bisulfite treatment, unmethylated cytosines are converted into uracil, followed by detection methods such as methylation-specific PCR (MSP), reduced representation bisulfite sequencing (RRBS), and methylation-sensitive high-resolution melting (MS-HRM).

Classic methods for detecting histones post-translational modifications (100) include chromatin immunoprecipitation followed by sequencing (ChIP-seq), Cut&Tag, and Edman degradation. Edman degradation, a low-throughput sequencing method, is generally limited to analyzing the N-terminal 50 amino acids of proteins and cannot detect multiple proteins simultaneously; it is used for analyzing chemically unmodified N-terminal α-amino acids. Immunosequencing methods cannot detect unknown modification sites. More widely used methods for histones post-translational modifications detection now include mass spectrometry (MS) and ELISA techniques.

Research methods for non-coding RNA mainly include transcriptome sequencing (RNA-seq), Northern blotting, fluorescence *in situ* hybridization (FISH), and RNA-binding protein immunoprecipitation (RIP-seq). Currently, Solexa high-throughput sequencing (101) is extensively used. This method can simultaneously analyze hundreds of millions of nucleotide fragments, offering low cost, high precision, and requires a small sample volume. Additionally, ACAT-seq, as a crucial tool for understanding chromatin states and epigenetic regulation, aids researchers in analyzing chromatin accessibility, chromatin structure and three-dimensional organization, and the association between epigenetic changes and diseases. An overview of these common epigenetic research methods, including their advantages, disadvantages, and applicability, is summarized in **Table 1**.

**Table 1:** Comparative summary of epigenetic research methods.

Serial Number	Method	Applicable Epigenetic Type	Advantages	Disadvantages	References
1	WGBs	DNA Methylation	Whole genome coverage; high resolution; quantitative and precise analysis of methylation levels and patterns at each CpG site	High cost; complex data processing; not suitable for time-sensitive studies	(102, 103)
2	MeDIP	DNA Methylation	High throughput; high specificity; low cost; suitable for various species and sample types, especially for CpG-rich (methylation) or specific methylation	Low resolution; presence of affinity bias; not suitable for other types of DNA modifications (e.g., hydroxymethylation) or specific methylation	(104, 105)
3	450k Chip	DNA Methylation	High throughput; high resolution; accuracy; covers most functional regions and key genes of the genome	Lacks coverage of unknown or specific regulatory regions; high cost; strict sample processing requirements	(106)
4	MSP	DNA Methylation	High specificity; high sensitivity; simple and fast	PCR bias; not suitable for unknown methylation sites or large-scale methylation analysis	(107)
5	RRBS	DNA Methylation	High resolution; low initial DNA amount requirement; low cost	PCR bias; complex data processing	(108, 109)
6	MS-HRM	DNA Methylation	High resolution; simple and fast; no primer design required	PCR bias; limited by methylation range; semi-quantitative	(110)
7	ChIP-seq	Protein Modification	Whole genome coverage; high resolution; discovery of new gene regulatory elements	High sample requirements; presence of enrichment bias	(111, 112)
8	Cut&Tag	Protein Modification	High resolution; simple, low cost; initial cell number can be as low as 50	Antibody specificity requirements; potential for background noise and non-specific binding	(113)
9	MS	Protein Modification	Comprehensive; high resolution	Limited by enzyme cleavage; expensive	(114)
10	ELISA	Protein Modification	High sensitivity; high throughput; simple, low cost	Risk of cross-reactivity; limited dynamic range; not suitable for small molecule analysis; requires high-quality antibodies	(115, 116)
11	Edman Degradation	Protein Modification	Efficiently determines the N-terminal sequence of proteins, suitable for small samples or high precision sequence information	Low throughput; time-consuming	(117)
12	RNA-seq	Non-coding RNA	Whole genome coverage; high throughput; detection of new genes and variants	Complex data analysis; high cost; high requirements for RNA integrity	(118, 119)
13	Northern Blot	Non-coding RNA	Can measure RNA size and relative abundance; shows RNA expression patterns and differential expression; quantitative	Low throughput; time-consuming; does not provide comprehensive transcriptome information	(120)
14	RIP-seq	Non-coding RNA	High throughput; high sensitivity; full transcriptome coverage	Relies on antibody specificity; not suitable for all RNA	(121)
15	FISH	Non-coding RNA	High resolution; can detect multiple targets simultaneously; maintains sample structure	Limited to static analysis; limited to known sequences	(122)
16	Solexa Sequencing	Non-coding RNA	High throughput; high sensitivity and accuracy; short time requirement	Complex data processing; relies on reference genome	(123)

#### 5.2.1 Environmental chemical substances

Heavy metal pollution has become a significant and widespread environmental issue that poses multiple hazards to human health. Cadmium, a typical heavy metal, exerts its reproductive toxicity, carcinogenicity, and other toxic effects by mediating changes in epigenetic modifications to regulate gene expression (268). Cadmium exposure can affect the overall level of genomic methylation. It leads to abnormal expression of DNMTs enzymes, with significant reductions observed in the mRNA and protein levels of DNMT1, DNMT3A, and DNMT3B, thereby inducing global DNA hypomethylation *in vitro (* 124). Similarly, cadmium exposure can also cause DNA hypermethylation to regulate gene expression. Aigner GP et al. (125), using earthworms as a model organism, revealed cadmium-induced hypermethylation of adenine and cytosine in spot imprinting, thus elucidating the time- and dose-dependent effects of Cd on global and gene-specific DNA methylation and its potential mechanisms.

Research has shown that continuous exposure of Drosophila melanogaster to cadmium during growth results in a significant increase in H3K4me3 levels and a significant decrease in H3K9me3 and H3K27me3 levels in third-instar larvae of the offspring. The expression of histone methylation-related genes dSet-1, ash1, and Lsd1 is significantly increased. Cadmium-induced wing phenotypic defects can be inherited by offspring, suggesting a potential transgenerational effect related to histone methylation’s epigenetic regulation (126).

Chen et al. (127)studied 31-week-old Hy-Line Brown hens fed with dietary cadmium chloride (150 mg/kg) and found a close association between miR-33 and cadmium toxicity. Cadmium toxicity significantly inhibited the expression of miR-33 and significantly increased the mRNA and protein levels of AMP-activated protein kinase(AMPK), altering the expression pattern of the miR-33-AMPK axis in the spleen and causing dysregulation of the miRNA-33-AMPK axis.

Mercury (Hg) is also a heavy metal that, due to its toxicity and widespread human exposure, has become a significant public health concern. Mercury is found in a variety of sources including seafood, household products, medical devices, and cosmetics, making it a common occupational hazard. Mercury pollution and volcanic eruptions are significant sources of its presence in soil, water, and the atmosphere. High doses of mercury are widely recognized as neurotoxic substances that negatively affect the female reproductive system (128).

Studies have shown that whole-body exposure to 2.5 mg/m³ of mercury for 6 hours per day over 6-8 weeks leads to prolonged estrous cycles in treated female rats and increased mortality rates in offspring (129). Davis et al. (130) found immature corpora lutea in rats exposed to mercury vapor. Lamperti et al. (131) observed inhibition of follicle maturation after injecting HgCl2 into hamsters. Dansereau et al. directly demonstrated that female minks exposed to a dietary concentration of 1.0 microgram/gram of mercury had fewer births compared to those exposed to 0.5 micrograms/gram and 0.1 micrograms/gram (132).

The theory of endocrine disruptors was initially proposed in the 1990s, identifying certain exogenous chemicals that interfere with the endogenous hormonal axis (133). These chemicals can interact in various ways within the body and affect different physiological areas. They include a variety of substances found in the environment, such as various pesticides, industrial chemicals (like Bisphenol A and phthalates), and dioxins. The presence and persistence of endocrine-disrupting chemicals (EDCs) in the environment impact organisms, and increasing evidence suggests that EDCs may be etiologically linked to the development and severity of diseases. The reproductive system is a primary target for most endocrine disruptors (134). Universal exposure during early development has been linked to the incidence of female cancers, especially reproductive organ cancers such as breast and ovarian cancer (135). In particular, *in utero* exposure might affect processes that initiate tumor growth years later. Additionally, some gynecological diseases are associated with exposure to various environmental toxins, particularly during critical developmental stages. Bisphenol A (BPA) was the first synthetic chemical found to cause selective estrogen receptor modulation, particularly as a hormone-like pollutant. Recent studies have shown that prenatal exposure to BPA might cause a phenotype similar to endometriosis in mice (136). Phthalates and BPA are chemicals widely present in many products, such as food packaging and household items. They are typical endocrine disruptors present in various substances and are frequently exposed to the public. Inhalation, ingestion, and skin contact are all possible exposure pathways (137). Urine analysis is a feasible method to confirm human exposure to these chemicals. Studies have shown that endometriosis is associated with elevated levels of phthalate metabolites and BPA metabolites in bodily fluids (138).

In the environment, pesticides are also endocrine disruptors. Current data indicate that approximately 2.5 million tons of pesticides enter the environment annually (139). Pesticides tend to accumulate in the environment due to their lipophilicity, long half-lives, and long mobility, causing significant environmental pollution (140). Pesticides can enter the human body through inhalation or skin penetration, but the highest toxicity is from ingesting contaminated food or water, including fish, meat and dairy products (especially the high-fat parts), drinking water, indoor and environmental air, and dust and soil (141, 142). Dichloro-Diphenyl-Trichloroethane(DDT), one of the most widely used pesticides, can bind with lipids and accumulate in adipose tissue (143). Long-term presence of pesticides in the human body can affect fertility and alter the levels of male and female reproductive hormones. These chemicals have anti-androgenic and estrogen-like properties (144), which may lead to stillbirth, birth defects, spontaneous abortions, and infertility. Animal studies suggest that daily exposure to DDT during prenatal and postnatal development could cause gender differences in steroid levels, possibly through direct interference by DDT and its impact on the hypothalamic-pituitary system (144). Research has also found that the disruptive effects of DDT involve competition with testosterone and damage to androgen receptor binding and signal transduction, as well as an association with increased estrogen synthesis (145). These changes in hormone secretion may be related to reproductive problems and physical diseases in later life, as excessive estrogen secretion and an imbalanced testosterone/estradiol ratio are associated with increased risks of feminization, metabolic disorders, estrogen-related cancers, and cardiovascular diseases (146).

Among the environmental pollutants that have been confirmed to promote transgenerational inheritance of epigenetic phenotypes, endocrine disruptors constitute a heterogeneous group of substances capable of interfering with hormone signaling pathways, directly altering germ cell epigenetic modifications, and changing metabolism and reproductive function (147). For instance, exposure to BPA increases DNA methylation and histone acetylation in zebrafish testicular cells (148). Male zebrafish exposed to BPA early in spermatogenesis and analyzed for F1 embryos showed increased histone acetylation induced by BPA, resulting in cardiac toxicity (149). Interestingly, research has identified three pairs of miRNA-mRNA involved in hypoxia-induced reproductive disorders, including novel miRNA-525-DIAPH2, novel miRNA-525-myocardium, and novel miRNA-525-RAI14, indicating for the first time that miRNAs may participate in hypoxia-induced reproductive disorders through transgenerational inheritance (147).

Furthermore, exposure to other endocrine disruptors such as organic compounds like benzo[a]pyrene (150) and air pollution particulate matter like CO2 (151) is associated with epigenetic changes, mediating DNA methylation, histones post-translational modifications, RNA expression alterations, and inducing human cancers and other diseases. These changes can be transgenerationally inherited and manifest as alterations in fertility, metabolic function, or behavioral traits.

Environmental pollution’s impact on human health is becoming increasingly apparent, with particulate matter (PM) pollution now recognized as one of the most critical public health risks. Particulate matter pollution encompasses various airborne particles, ranging in size from a few micrometers to visible particles up to 100 micrometers. Long-term exposure to environmental particulate matter can lead to cardiac and pulmonary diseases. Most studies focus on particulate matter with an aerodynamic diameter less than 10 micrometers (PM10) or less than 2.5 micrometers (PM2.5), which may adversely affect fetal development, the normal course of pregnancy, and lead to premature birth (152).

Research indicates that high concentrations of PM10 are closely associated with an increased risk of pregnancy complications throughout the gestation period and its various stages. Particularly during the late stages of pregnancy, exposure to high levels of PM2.5 significantly increases the risk of pregnancy complications. In the middle stage of pregnancy and throughout the entire gestational period, every 10 μg/m³ increase in PM10 concentration increases the risk of preterm birth (PTB) by 24% and 27%, respectively. Additionally, exposure to high concentrations of PM10 during the mid-pregnancy stage increases the risk of gestational diabetes mellitus (GDM) by 30%. For PM2.5, every 5 μg/m³ increase in concentration raises the risk of GDM by 15% in the mid-pregnancy stage and 25% throughout the entire pregnancy. In the first three months of pregnancy, exposure to high concentrations of PM10 and PM2.5 increases the risk of having a small for gestational age (SGA) infant by 96% and 26%, respectively (153).

#### 5.2.2 Other factors

There are reports indicating a positive correlation between the highly prevalent sexually transmitted protozoan parasite *richomonas vaginalis* and vaginal and cervical neoplasms in women, as well as prostate cancer in men (154). Infection with *T. vaginalis* significantly alters the structure of the vaginal microbiome, shifting from a lactobacilli-dominated community to one that favors the widespread transmission of bacterial vaginosis (155). This parasite releases metabolites, such as indoles, which aid in the survival of intracellular spreading bacteria like *Chlamydia trachomatis*, which has been independently associated with cancer (156). Given the positive correlation between bacterial vaginosis and precancerous lesions of the cervix (157), it is necessary to conduct research to clarify the role of the microbiome in *T. vaginalis -associated* vaginal carcinogenesis, whether as a cofactor or a necessary factor.

**Figure 2:**
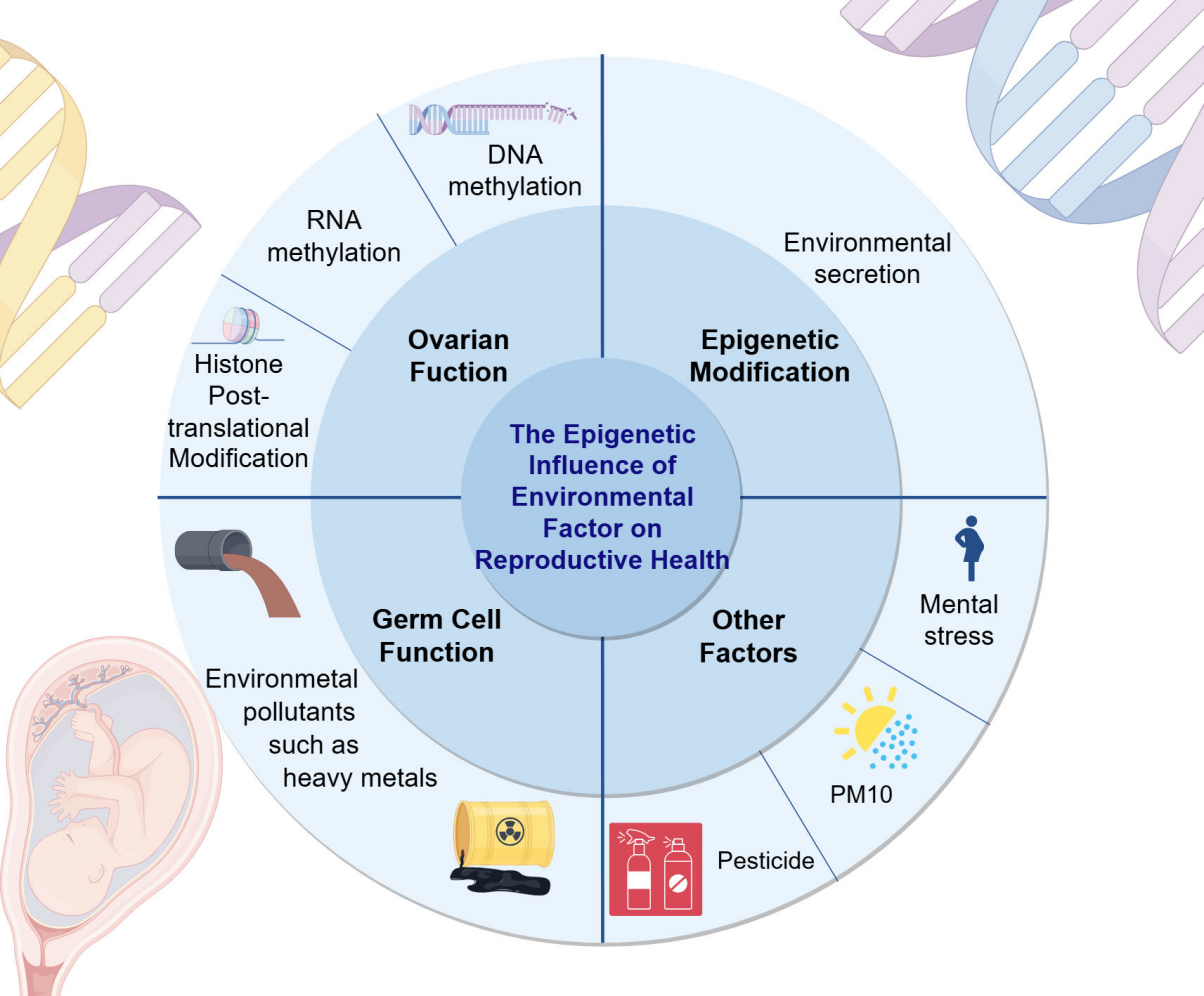
The epigenetic impact of environmental factors on reproductive health. This figure elucidates the influence of environmental factors on reproductive health from three aspects: ovarian function, epigenetic modifications, germ cell function, and other examples are given to illustrate the impact of environmental factors on reproductive health. For example, the negative effects of PM10 and pesticides on women’s reproductive health. (Draw by figdraw.).

Climate factors such as global warming (158) can also lead to transgenerational transmission of specific histones post-translational modifications, DNA methylation modifications, and other epigenetic marks. This article provides a concise summary of common environmental factors along with their epigenetic impacts and mechanisms of action, as presented in **Table 2** and **Figure 2**.

**Table 2:** Environmental factors that can induce epigenetic changes affecting female reproduction.

Environmental Factors			Reproductive Impact	Involved Epigenetic Mechanism	References
Heavy Metals	Cadmium		Endometriosis, uterine fibroids, miscarriage	Activates certain cellular signals, suppresses DNA methylation, increases miR-146a expression	(159)
	Lead		Breast cancer, heart disease	Gene-specific hypomethylation	(160, 161)
Endocrine Disruptors	Bisphenol A		Breast cancer, oocyte development defects	Induces DNA hypomethylation, increases miR-146a overexpression	(162, 163)
	Phthalates		Uterine fibroids, endometrial hyperplasia	Induces DNA hypermethylation	(164, 165)
	Pesticides	DDT	Ovarian tumors, polycystic ovary syndrome, endometriosis, infertility	Affects DNA methylation	(166)
		Atrazine	Pregnancy complications, anemia, breast cancer	Gene-specific CpG methylation changes, affects gene expression, chromatin remodeling, and DNA methylation	(167)
Environmental Pollutants	PM10, PM containing heavy metals		Gestational diabetes, preterm birth, low birth weight, stillbirth, birth defects	Induces gene expression changes, rapid changes in miR-21 and miR-222 expression	(168, 169)
	Chemicals		Infertility, decreased ovarian reserve, uterine fibroids, ovarian cancer	Affects microRNA expression and regulation	(170)

### 5.3 The regulation of female reproductive function by epigenetics

#### 5.3.1 Ovarian function and epigenetic regulation

PCOS, as a heterogeneous disease leading to reproductive and metabolic disorders in women, is the most common cause of infertility in women of childbearing age (171). A study (172) analyzed the DNA methylation in ovarian tissues of PCOS-like mice, and these findings suggest that PCOS can be transmitted to offspring through changes in DNA methylation in epigenetics, proposing that methylation biomarkers may serve as potential diagnostic indicators for this disease. Another study indicated that the histone deacetylase inhibitor valproic acid can reduce metabolic dysfunction in the skeletal muscles of PCOS rats by inhibiting PDK4/NLRP3 inflammasome (173). Therefore, inhibiting histone acetylation may aid in the diagnosis and treatment of PCOS. Additionally, alterations in non-coding RNAs (ncRNAs) are one of the mechanisms of PCOS (174). In recent years, numerous studies have shown significant abnormalities in the expression of ncRNAs in follicular fluid, serum, ovarian granulosa cells (175), and other tissues of women with PCOS. Therefore, analyzing the abnormal expression of ncRNAs in PCOS patients can serve as diagnostic biomarkers and play crucial roles as therapeutic targets in the treatment of PCOS.

**Figure 3:**
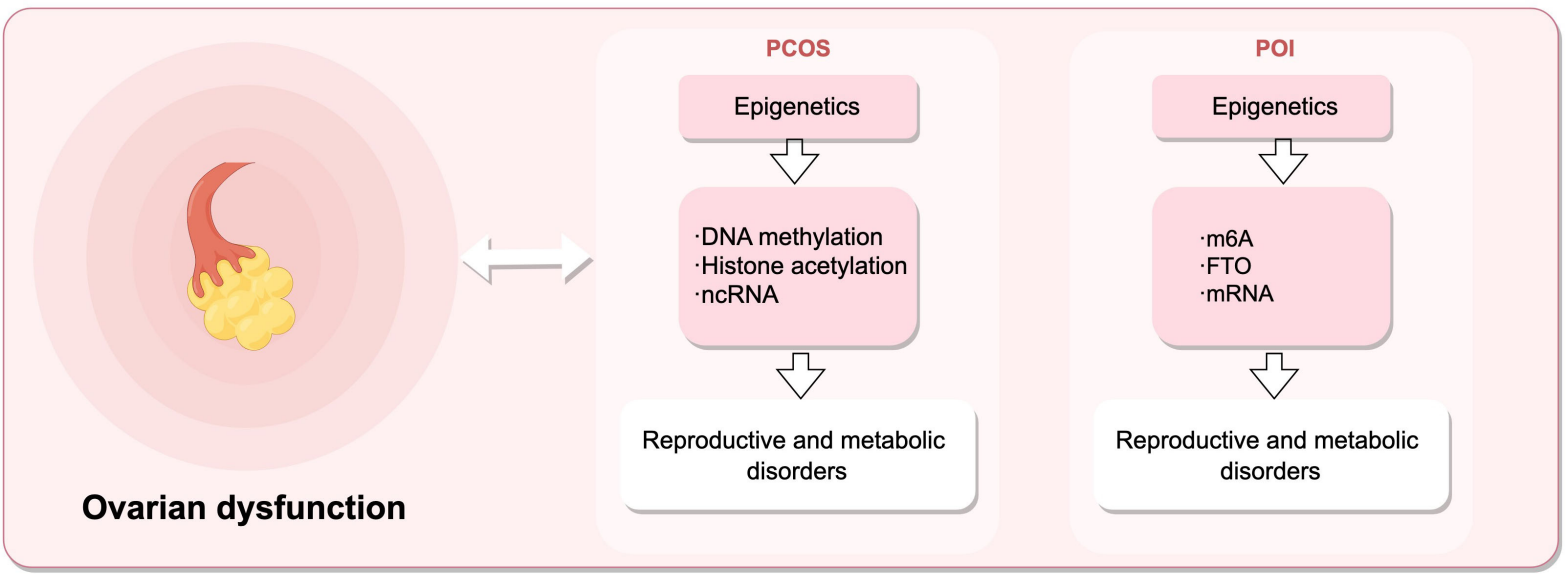
Epigenetic mechanisms regulate female reproductive capacity, leading to ovarian dysfunction. Dysregulation of DNA methylation, histones post-translational modifications, and ncRNAs can result in polycystic ovary syndrome (PCOS), while alterations in m6A, FTO, and mRNA levels contribute to premature ovarian insufficiency (POI). (Draw by figdraw.).

As shown in **Figure 3**, premature ovarian insufficiency depicts common ovarian dysfunctions and their epigenetic mechanisms. POI refers to the ovarian function decline characterized by elevated gonadotropins and estrogen deficiency in women before the age of 40, mainly manifested as menstrual abnormalities, ultimately progressing to premature ovarian failure (POF) with varying degrees of perimenopausal symptoms (176), affecting the fertility and quality of life of women of reproductive age. N6-methyladenosine (m6A) modification can effectively regulate the epigenetics of mammalian transcriptome. A case-control study (177) measured the m6A content in the RNA of POI patients and controls. Compared with the control group, the m6A content in the granulosa cells of POI patients was significantly increased, accompanied by a significant decrease in FTO mRNA and protein expression levels. The results indicate a strong association between m6A content and the risk of POI, which may impair ovarian function and further lead to complications of POI.

#### 5.3.2 Fertility capability

In non-disease conditions, epigenetics can influence the female reproductive environment, thus affecting fertility. The proper development and maturation of oocytes not only directly impact fertility but also relate to embryo health and the likelihood of successful pregnancy. Appropriate methylation of oocytes is crucial for coordinating gene expression patterns, driving oocyte developmental programs, and ensuring oocyte quality. N6-methyladenosine (m^6^A) is the most common internal modification of mRNA (178), playing a role in oocyte maturation. Specific knockout of METTL3 in Gdf9-Cre mouse oocytes disrupts normal mRNA methylation, leading to DNA damage, follicular development defects, and ovulation abnormalities. It has been shown that the methyltransferase METTL3 may enhance the stability of m6A modifications on Itsn2, affecting oocyte meiosis (179, 180). Similarly, studies have found that the negative mutant H3.3-K4M specifically expressed in mouse oocytes reduces H3K4 methylation levels, leading to decreased transcriptional activity and increased DNA methylation in oocytes, disrupting oocyte development and female mouse fertility. Early embryos from H3.3-K4M oocytes exhibit developmental arrest and reduced activation of the zygotic genome (181).

Epigenetics also influences the role of hormones in the reproductive system by regulating gene expression, including hormone synthesis, secretion, and receptor sensitivity, thus affecting ovarian function, the uterine environment, and cyclic changes. Research has shown that the estrogen receptor α (ERα) can recruit various coregulators (such as histone modifiers, transcription factors, or other auxiliary proteins), where histone modifiers alter the chromatin structure and organization, regulate gene accessibility and transcriptional activity, and promote chromatin opening, facilitating the binding of ERα and transcription mechanisms to estrogen response elements (ERE) (182). SMYD2 is a proven negative regulator of ERα, primarily inhibiting the activation of estrogen receptor target genes by aiding methylation of ERα protein at the K266 site (183). An epigenetic axis exists between TET2 and ERα, with endogenous TET2 occupying active enhancers and promoting proper recruitment of ERα. DNA demethylation activates enhancers to coordinate transcription programs, enhancing estrogen response (184). Besides influencing reproductive hormone receptors, epigenetics can cause changes in reproductive hormone synthesis. Endometriosis or adenomyosis cells often exhibit aberrant epigenetic programming mechanisms. Binding of NR5A1 to the proximal promoter of the CYP19A1 gene may promote demethylation of the NR5A1 gene promoter region, leading to overexpression of estrogen receptor-b (ESR2), excessive estrogen formation, abnormal interaction of estrogen with ESR2, and progesterone resistance (185). In regulating the internal hormonal environment in women, the hypothalamus serves as a key hub of the endocrine system, regulating the secretion of multiple hormones. By secreting gonadotropin-releasing hormone (GnRH), it controls the anterior pituitary secretion of follicle-stimulating hormone (FSH) and luteinizing hormone (LH) (186). Epigenetic mechanisms regulate the expression of related hypothalamic genes, affecting the synthesis and secretion of GnRH, thereby controlling the release of FSH and LH, stimulating follicular development and ovulation, thus maintaining a normal reproductive cycle (187). A study showed that perinatal exposure to EDCs can reprogram DNA methylation and steroid hormone receptor expression through epigenetic mechanisms to regulate female fertility (188).

When embryos develop to the blastocyst stage, they can enter the uterine cavity and interact with the endometrium, a process regulated by epigenetics. During this process, the miRNA processing enzyme Dicer is upregulated, and microRNA Let-7a is downregulated, enabling the blastocyst to acquire the ability to implant in the uterus (189). During embryo implantation, DNA methylation can regulate the expression of HOXA10. Abnormal DNA methylation can downregulate HOXA10 expression (190), affecting endometrial receptivity. Targeted destruction of HOXA10 in female mice can lead to embryo death and implantation failure (191). Endometrial angiogenesis is a necessary condition for good endometrial receptivity, and a study showed that the KLF4 (Krüppel-like factor 4)-VEGFA (vascular endothelial growth factor A) positive feedback loop is regulated by epigenetics, promoting proliferation and migration of human endometrial microvascular endothelial cells (HEMECs), inhibiting apoptosis, thus enhancing endometrial receptivity (192). Abnormal expression of non-coding RNA miRNAs can lead to defects in human endometrial receptivity. Patients with recurrent implantation failure (RIF) in IVF exhibit lower mRNA levels of cell adhesion molecules, Wnt signaling components, and cell cycle pathways, including N-cadherin, H2AFX, netrin-4, and secreted frizzled-related protein-4 (193).

**Figure 4:**
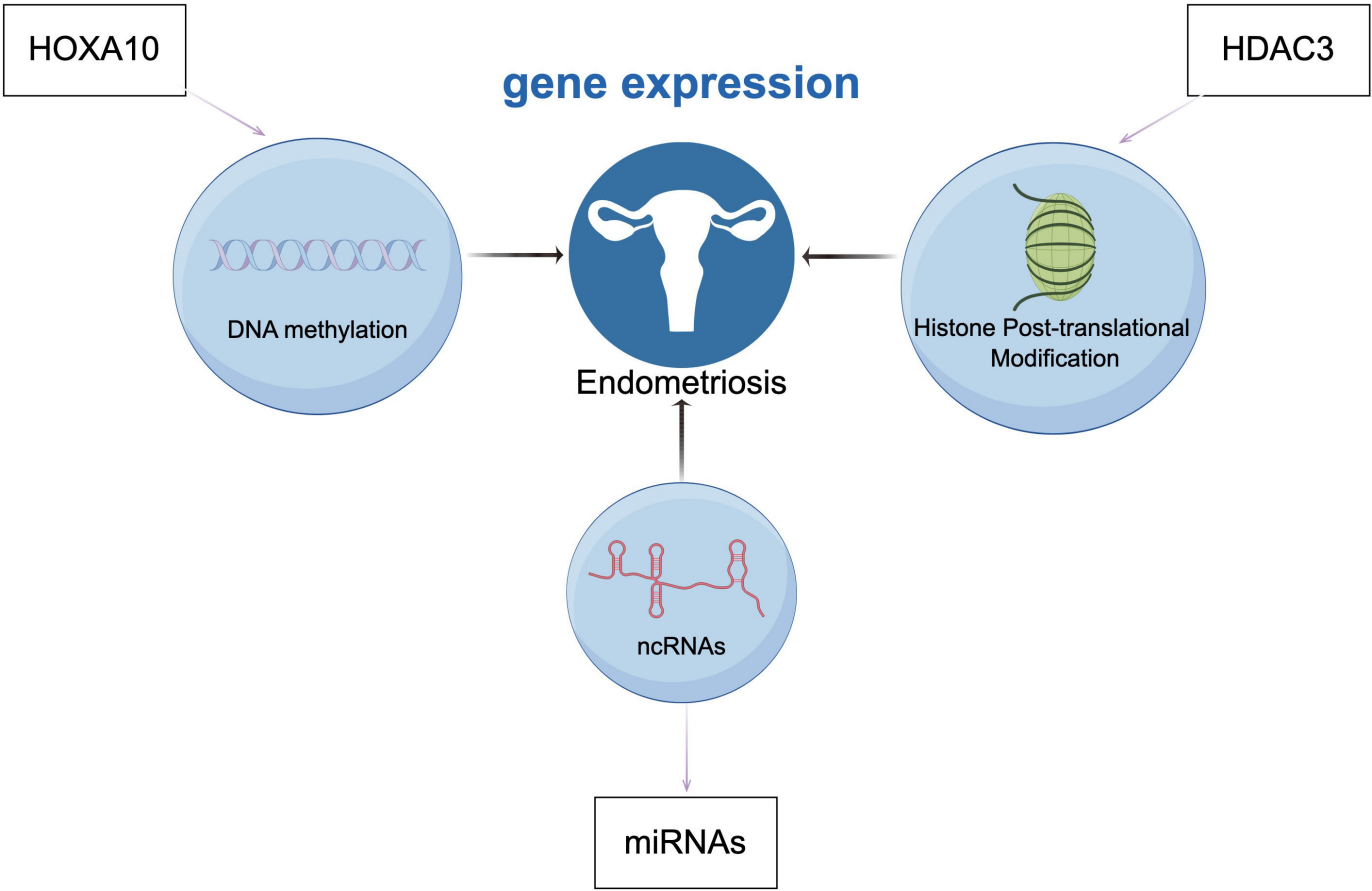
Endometriosis is associated with epigenetic mechanisms related to gene regulation, DNA methylation (HOXA10), histones post-translational modifications (HDAC3), and non-coding RNAs (ncRNAs). (Draw by figdraw.).

Endometriosis is a common benign inflammatory gynecological disease characterized by the presence and growth of endometrial-like glands and stroma outside the uterus, leading to pelvic pain and reduced fertility in reproductive-aged women, significantly impacting the quality of life of affected women 194. Epigenetic processes can regulate gene expression during endometrial development throughout the menstrual cycle through various mechanisms, altering the function and morphology of the endometrium. Epigenetic dysregulation plays an important role in the pathogenesis and pathophysiology of endometriosis, with aberrant expression of epigenetic processes found in the endometrium of affected women, holding great potential as therapeutic targets, diagnostic, and prognostic markers (195). DNA methylation is one of the most common epigenetic modifications in endometrial biology, with changes in DNA methylation occurring during different stages of the menstrual cycle (196). As a representative example, high methylation of the HOXA10 gene promoter in the endometrium of women with endometriosis leads to gene silencing, resulting in decreased levels of HOXA10 in the ectopic endometrium, potentially impairing female fertility (197). Similarly, histones post-translational modifications are associated with endometrial function. Reduced protein levels of histone deacetylase 3 (HDAC3) in the ectopic endometrium of infertile women with endometriosis may impair fertility, as HDAC3 is crucial for endometrial receptivity and decidualization. As a typical example, studies by Samartzis, E.P. et al. on the impact of Hdac3 deletion in mouse uteri on fertility demonstrated that Hdac3 deficiency can lead to aberrant transcriptional activation of two direct targets of mouse and human HDAC3, COL1A1 and COL1A2(COL1A1 and COL1A2, genes that encodes the human collagen Iαchain), resulting in implantation and decidualization abnormalities and consequent loss of fertility (198). Thus, aberrant expression of HDACs can better explain the causes and mechanisms of endometriosis. A study evaluating differential expression of microRNAs in serum cultures of severe endometriosis patients and controls found significant dysregulation of six microRNAs. This study confirmed the role of miRNAs in the pathogenesis of endometriosis, demonstrating that serum-derived eMSCs from severe endometriosis patients can induce abnormal expression of miRNAs and their target genes, leading to the development of endometriosis (199). **Figure 4** illustrates the common epigenetic mechanisms and associated genes involved in endometriosis.

Pre-eclampsia is a multisystem disease broadly affecting pregnancy, annually responsible for over 60,000 maternal deaths globally and causing more than 500,000 cases of preterm birth (200). Studies have established an association between the ACVR2A gene and pre-eclampsia. Although single nucleotide polymorphisms (SNPs) related to the ACVR2A gene do not reside within its coding region, and thus do not directly alter the coding sequence of the ACVR2A protein, they may still influence the transcription of the ACVR2A gene by affecting the binding of transcription factors or the regulation by microRNAs. ACVR2A is located on chromosome 2q22, and its encoded type II activin receptor binds the activin A ligand (201). In maternal tissues, activin A promotes decidualization of the endometrial stroma cells and regulates the invasion process of the trophoblast (202); in fetal tissues, it is essential for trophoblast differentiation, placental development, and the functional regulation of the trophoblast (203).

Adenomyosis, a common gynecological disorder in women of reproductive age, is characterized by the aberrant invasion of endometrial tissue into the myometrium and is closely associated with infertility (204). Studies on the expression and localization of DNMT in healthy females and those with adenomyosis revealed heightened immunoreactivity to DNMT1 and DNMT3B in ectopic endometrium (265), whereas DNMT3A staining levels significantly decreased in both eutopic and ectopic endometrium. Beyond DNA methylation, abnormal expression and localization of class I histone deacetylases (HDACs) in the endometrium have also been confirmed (205). Additionally, lower total m6A levels in the myometrium of patients with adenomyosis have been linked to differential expression of METTL3 and ZC3H13 (206), suggesting that m6A RNA methylation regulatory factors may participate in the pathogenesis of adenomyosis through aberrant expression in the endometrium.

Furthermore, improper maintenance of heritable epigenetic markers can result in aberrantly activated or suppressed signaling pathways, leading to malignant tumors in the reproductive system (207). Research has shown that epigenetic modifications like DNA methylation can influence TMB and play an indispensable role in tumor onset, as proposed by Liu B et al., where DNA methylation in the tumor microenvironment (TME) affects the expression levels of certain cervical cancer genes, further influencing the immune response in the TME, thereby inducing cervical cancer (208). Moreover, other reproductive system cancers such as endometrial cancer, ovarian cancer, and uterine fibroids can also arise due to epigenetic induction, leading to infertility and life-threatening conditions for the patient.

Uterine fibroids (also known as leiomyomas), the most common benign gynecological tumors among women of reproductive age worldwide, can cause severe anemia, pelvic pain, and infertility by affecting the overall function of the endometrium (209). Compared to normal myometrial layers, uterine fibroids exhibit altered levels of DNA methylation, increased estrogen receptor mRNA, and DNA methyltransferase levels. The activation of the PANKL gene plays a crucial role in the development of uterine fibroids by activating stem cells in the myometrial layer (210), while DNA methylation and MED12 gene mutations form a complex regulatory network affecting the expression of the PANKL gene mediated by progesterone and its receptors. Furthermore, post-translational modifications of histones in uterine fibroid tissues have changed, particularly through genomic activation-related histone acetylation; acetylated histone H3K27 is involved in regulating genes related to cell signaling, transport, angiogenesis, and extracellular matrix formation, promoting the development of uterine fibroids (211). The miR-29 family is associated with the deposition of the extracellular matrix, and the miR-3 family can regulate cyclins, also interacting with long non-coding RNAs (lncRNAs) to promote the production and deposition of the extracellular matrix, providing a physical platform for tumor cell growth, migration, and spread. Overproduction of extracellular matrix components not only increases the hardness and volume of the fibroids but also regulates the TGF-β signaling pathway through miRNA expression, thereby affecting the normal biological functions of the uterine wall, such as menstrual bleeding, fertility, and embryo implantation. In this context, research by Włodarczyk et al. (212) further indicates that abnormal changes in TET protein and 5hmC levels may be key factors leading to the formation of uterine fibroids. Additionally, lncRNAs can directly activate the Wnt/β-catenin pathway through the estrogen receptor, promoting the proliferation of uterine fibroid cells (213).

Ovarian cancer poses a serious threat to female reproductive health, with miRNAs playing a critical role in the pathogenesis and progression of the disease (214). Studies have found that certain miRNAs are aberrantly expressed in ovarian cancer tissues, showing upregulation or downregulation, directly affecting multiple key biological processes including cell proliferation, apoptosis, metastasis, and invasion capabilities (215). For instance, the significant downregulation of miR-126-3p in ovarian cancer cells is closely related to tumor cell proliferation and invasion (216). In the treatment process, aberrant miRNA expression is also associated with resistance of ovarian cancer cells to chemotherapy drugs, potentially leading to disease relapse and progression (217). Additionally, single-gene methylation biomarkers such as RASSF1A and BRCA1 are significant factors in the onset and progression of ovarian cancer. Studies have shown that high methylation levels of BRCA1 and RASSF1A are prevalent in ovarian cancer patients, with 82% of patients exhibiting the same high methylation pattern in serum or plasma BRCA1 and RASSF1A (218). Methylation of these genes is highly correlated with clinical features of ovarian cancer such as FIGO stage, plasma CA-125 levels, and histological type (219).

In the development of cervical cancer (CC), persistent HR-HPV infection, aberrant methylation of the host cell genome and HPV genome DNA in cervical squamous epithelial cells can lead to dysfunction of various tumor suppressor genes, promoting the development of CC (220). The cervix, a critical gateway for nurturing life, is highly sensitive to estrogens, and research in an HPV transgenic mouse model has shown that estrogens and their nuclear receptors, in conjunction with HPV oncogenes, promote the onset of cervical cancer (221). Highly methylated and lowly expressed genes (Hyper-LGs) are significantly enriched in estrogen receptor pathways and the Wnt/β-catenin signaling pathway, affecting estrogen expression (222). Current research on post-translational modifications of CC proteins has focused on histone acetylation. One study showed that histone H3 and H4 acetylation is associated with the activation of HPV16 gene expression levels in CC cells, with histone acetylation levels increasing as HPV16 gene expression increases, thereby advancing the development of CC (223). Moreover, extensive research has demonstrated that lncRNAs are involved in the malignant transformation of cervical epithelial cells. For example, lncRNAMIR210HG is overexpressed in CC tissues and promotes cell proliferation and invasion through hypoxia-inducible factor-1α (HIF-1α) (224).

The endometrium, a hormone-sensitive tissue, undergoes numerous biochemical and morphological changes during the normal menstrual cycle under the control of steroid hormone levels, while abnormal exposure to estrogens can significantly increase the risk of endometrial cancer (225). DNA methylation can affect the functional changes of endometrial tissue, for instance, overall DNA methylation status and progesterone receptor levels are significantly increased during the proliferative phase and decrease at the end of the secretory phase (226). Apoptotic cell death mediated elimination of senescent cells in the functional layer of the endometrium helps maintain cellular homeostasis, thus avoiding apoptosis remains a major issue in the successful treatment of late-stage endometrial cancer. Abnormal changes in DNA methylation can lead to the deregulation of key apoptotic proteins during the development of endometrial cancer, resulting in the development of apoptosis resistance (227).

#### 5.3.3 Specific studies

Women exposed to various chemical, biological, physical, and sociopsychological factors may experience impacts on their reproductive systems. These effects can manifest as changes in sex hormone levels, sexual dysfunction, menstrual disorders, early menopause, delayed menarche, impaired ovarian function, reduced fertility, and adverse pregnancy outcomes. During pregnancy, maternal exposure can disrupt normal fetal development, such as intrauterine growth retardation, preterm birth, birth defects, and impacts on cognitive development and immune function (228). Thakur et al.’s study showed that in areas affected by heavy metals and pesticides, the rate of spontaneous abortions is 20.6 per 1000 live births, and the rate of preterm births is 6.7 per 1000 live births, significantly higher than in non-polluted areas. Petrelli et al. (229) found that the abortion/pregnancy ratio for pesticide applicators was 0.27, compared to 0.07 for food retailers. In a multivariable logistic regression model, after adjusting for the wife’s age and parents’ smoking habits, the odds ratio for spontaneous abortion was 3.8 times higher compared to the control group; considering interaction effects, this ratio increased to 7.6 times. Both men and women exposed to certain pesticides face increased risks of abnormal sperm, reduced fertility, increased spontaneous abortions, male birth defects, birth defects, or fetal growth retardation (230). Logan and Chen (231) noted that exposure to bis(4-chlorophenyl)-1,1,1-trichloroethane (DDT) might reduce the rate of preterm births, thereby lowering infant mortality in malaria control. Additionally, Salazar-García et al. (232) reported that occupational exposure to DDT is associated with an increased risk of birth defects. It has been reported that levels of DDT metabolites (p, p’-DDE) are higher in 100% of infertile women. Furthermore, studies (233) have also found that pesticide exposure can lead to reduced fertility. Moreover, parents working in agriculture may increase the risk of congenital anomalies in their children, such as hemangiomas, orofacial clefts, neurological damage, and musculoskeletal defects.

In conclusion, environmental factors can induce epigenetic changes that affect female reproductive function. Epigenetics serves as a bridge and mediator, establishing a connection between environmental factors and female reproductive function. Below are specific research examples. A cross-sectional study based on 1647 American women aged 20-54 with endometriosis from 1999 to 2006 showed a positive correlation between urinary cadmium levels and the prevalence of endometriosis (234). Additionally, research exposed fruit flies to cadmium during egg development to adulthood, then cultured the offspring in a cadmium-free environment. Under cadmium exposure, the expression of histone methylation-related genes significantly increased in the ovaries of third instar larvae and adult flies, with a marked increase in histone H3K4me3 post-translational modification and a decrease in H3K9me3 and H3K27me3 levels. These changes could be transmitted to the offspring’s ovaries, leading to changes in reproductive ability (126). Female reproductive capacity begins in fetal ovaries, and early steps in folliculogenesis are sensitive to environmental factors. The quality of oocytes is closely linked to the process of folliculogenesis, with a long window of susceptibility to environmental damage. After fertilization, fertilized eggs and pre-implantation embryos undergo extensive epigenetic reprogramming. The FEDEXPO project studied potential transgenerational inheritance based on epigenetic markers in F1 offspring gametes, demonstrating that early and perinatal environments can have adverse effects on female reproductive capacity. Abnormalities in epigenetic processes and imprints may affect the health of future generations (235). Therefore, it is crucial to closely monitor and assess the toxicity and risks of environmental factors, avoid inducing epigenetic changes that may harm female reproductive capacity, and consider the adaptability of future generations.

### 5.4 The role of epigenetics in female reproductive health

In the field of female reproductive health, epigenetic processes such as DNA methylation and histones post-translational modifications play a pivotal role. These changes are crucial in the transition from the maternal environment of the oocyte to the embryo-driven developmental expression program (236), thereby significantly influencing the regulation of ovarian function, oocyte maturation, and embryo development.

DNA methylation, particularly in the regulation of ovarian function, plays a key role. Alterations in DNA methylation can affect the activity of specific genes, thereby regulating the levels of hormones in the ovaries, which are vital for fertility. For instance, methylation changes in genes that affect oocyte maturation and the ovulatory cycle may lead to fertility-related issues.

Taking m6A as an example, N6-methyladenosine, also known as m6A, is a widely occurring base modification on mRNA and represents the most prevalent form of RNA modification in the human body. Due to the dynamic and reversible regulation of various biological processes by m6A (237), abnormal increases or decreases in its levels can lead to the occurrence of different diseases, including many female reproductive endocrine disorders such as endometriosis, polycystic ovary syndrome, and malignancies of the female reproductive system like endometrial cancer and cervical cancer (172, 198, 238).

In the development of these diseases, DNA methylation and m6A modifications may exert their effects through pathways such as influencing the expression of specific genes or regulating hormone levels in the ovaries. These findings underscore the importance of in-depth research into the role of epigenetics in female reproductive health to better understand, prevent, and treat related diseases.

#### 5.4.1 The epigenetic impact of environmental factors on reproductive health

Environmental factors such as diet, lifestyle, psychological stress, and exposure to chemicals play a significant role in female reproductive health. These factors can alter patterns of DNA methylation and histones post-translational modifications, affecting gene expression and thereby significantly impacting reproductive health.

For instance, long-term exposure to certain environmental pollutants, such as heavy metals, organic pollutants, and endocrine-disrupting chemicals, may lead to changes in the epigenetic markers of eggs and sperm (239, 240). These changes can affect the functionality of reproductive cells, such as the maturation of oocytes and the vitality of sperm, thereby impacting the healthy development of embryos and fertility.

Studies have shown that environmental endocrine disruptors may affect epigenetic modifications, interfering with the development of reproductive cells (241).

Increasing evidence suggests that widely prevalent environmental pollutants known as endocrine-disrupting chemicals (EDCs), such as BPA, polychlorinated biphenyls (PCBs), and phthalates, negatively impact reproductive health in animals and humans and are associated with various diseases, including infertility. EDCs can exhibit estrogen-like activity, mimicking or blocking the actions of endogenous hormones and affecting related genes, thus altering phenotypes. Hormones cause developmental changes in offspring through embryonic methylation and maintain these changes in germ cells. Evidence indicates that exposure to EDCs impacts female reproductive potential, as measured by ovarian reserve and outcomes of assisted reproductive technologies (ART) (242).

In addition to natural pregnancies, women undergoing ART are also susceptible to environmental influences. Studies have examined women who underwent ART and were closely monitored during early pregnancy to explore the association between exposure to types of environmental air pollution and the timing of miscarriages. It was found that higher NO2 exposure was associated with an increased risk of miscarriage within 30 days after a positive Human Chorionic Gonadotropin(HCG) test (243). Beyond miscarriages, immediate maternal complications related to ART are among the more apparent and recognizable issues, such as ovarian hyperstimulation syndrome (OHSS), and risks associated with ART procedures including egg retrieval, embryo transfer, and fetal reduction surgeries (244). Additionally, a range of derived issues such as risks associated with operative anesthesia, endometrial biopsy, and hysteroscopy can impact the individual and potentially affect the offspring.

Despite ART being a core technology for treating infertility in contemporary settings, a woman’s reproductive potential still largely depends on the quality of her oocytes and the maternal environment that supports embryo implantation and development. The influence of the perinatal environment on the epigenetics of developing embryos has become a focal point in research into the effects of the environment, nutrition, and assisted reproductive technologies on human development and health (245).

These findings indicate that environmental factors and lifestyle choices are crucial for maintaining reproductive health, especially in preventing and managing reproductive issues related to the environment. Therefore, understanding how environmental factors influence reproductive health through epigenetic mechanisms is essential for developing effective prevention and intervention strategies.

#### 5.4.2 Epigenetics and female fertility

Moreover, the impact of epigenetic changes on female fertility is a significant area of research. As age increases, the quantity and quality of eggs in the ovaries gradually decline, a phenomenon that may be related to changes in the methylation patterns of specific genes. Age-related changes in the methylation patterns of genes in eggs may affect their maturation process and fertilization capability. The decline in egg quality is not only related to epigenetic changes but also involves abnormalities in mitochondrial function, mutations in nuclear DNA, shortening of telomeres, misalignment of chromosomes, and inactivation of the spindle checkpoint. These changes may lead to early pregnancy loss, neonatal death, or chromosomal aneuploidy genetic diseases such as Down syndrome (246). In terms of epigenetic regulation, METTL3 is a key factor. Knocking out METTL3 severely inhibits the maturation of oocytes, affecting the transition from oocyte to zygote (247). Although the pathogenesis of most patients with ovarian dysfunction is not entirely clear, related experiments indicate (248) that the development of oocytes in Zmettl3m/m zebrafish (a zygotic defect mutant line targeting METTL3 exons) is delayed, with most remaining at an early stage. The m6A modification evidently affects the maturation rate of follicles; the experiment also pointed out that due to the significant reduction in m6A content in the oocytes of Zmettl3m/m zebrafish, the key factors related to *in vivo* sex hormone synthesis and gonadotropin signaling cannot be normally expressed. Consequently, this leads to a decrease in the secretion of 11-ketotestosterone and 17β-estradiol in the offspring embryos, ultimately causing gamete maturation disorders and reduced fertility. Additionally, the methylation status of some genes may affect the cell cycle regulation and aging of the oocyte (249), thereby impacting its maturation and quality.

#### 5.4.3 The role of epigenetics in pregnancy and embryonic development

During pregnancy and embryonic development, epigenetics plays a crucial role. The nutritional status of pregnant women, environmental exposure, and psychological state can influence the development of the embryo by altering its epigenetic status (250). These factors not only have a significant impact on embryonic development during pregnancy but may also have a profound effect on the long-term health of the offspring, including their metabolic status and disease susceptibility in adulthood.

Epigenetics plays a vital role at every stage of embryonic development. For example, during the early stages of embryonic development (251), the nutrition and environmental factors provided by the mother can regulate the gene expression and development of the embryo by affecting gene methylation and histones post-translational modifications. This epigenetic regulation is not only crucial for the normal development of the embryo but may also affect the long-term health and disease susceptibility of the embryo.

Moreover, the regulation of gene expression during the early stages of embryonic development is highly complex and dynamically ordered, supported by various factors, including transcription factors and epigenetic information such as chromatin accessibility, DNA methylation, and histones post-translational modifications. Transcription factors and epigenetic information (such as chromatin accessibility, DNA methylation, histones post-translational modifications, etc.) are important factors in the regulation of gene expression during early embryonic development (252, 253). These factors work together to ensure the smooth progression and transition of important biological processes at various stages of early embryonic development.

Therefore, in-depth research into the role of epigenetics in pregnancy and embryonic development is of great significance for understanding the mechanisms of normal embryonic development, predicting and preventing developmental abnormalities, and improving the health of offspring.

#### 5.4.4 The epigenetic effects of environmental factors on offspring health

Embryonic development and infancy are two critical periods that are particularly sensitive to environmental factors. During these stages, epigenetic programming is highly susceptible to various environmental factors such as diet, temperature, environmental toxins, maternal behavioral habits, and even childhood experiences of abuse. The epigenetic modifications of imprinted genes induced by these factors may lead to poor development of multiple organs in the fetus and may increase the risk of various diseases in adulthood (254). For instance, Professor Li Jingwen from Fujian Medical University has pointed out that exposure to cadmium during pregnancy in rats has shown effects on the regulation of miRNA and DNA methylation patterns in the offspring’s ovarian granulosa cells, revealing the potential for epigenetic changes to be inherited across generations. This cross-generational impact highlights the significance of environmental factors in affecting the health of offspring, especially how exposure to harmful substances during early pregnancy can have long-lasting effects on the health of the offspring.

The intrauterine environment and the early environment of newborns can provoke permanent responses in fetuses and newborns, thereby increasing their susceptibility to diseases later in life (255). Today, the mode of conception (such as *in vitro* fertilization), maternal metabolic conditions (such as malnutrition, overnutrition, diabetes), and pregnancy complications (such as preeclampsia and intrauterine growth restriction) are suspected to be negative predictors of long-term health in offspring.

Moreover, as ART become more widespread, while they compensate for familial deficiencies, they also bring subsequent health risks to offspring. During ART, significant epigenetic reprogramming occurs, which is crucial for the normal destiny of the embryo. This epigenetic reprogramming is highly susceptible to changes in environmental conditions, such as those inherent in *in vitro* fertilization, including *in vitro* culture, nutrients, lighting, temperature, oxygen tension, embryo-maternal signaling, and the general lack of protection against exogenous elements that could destabilize this process (256). Professor Zhuan Ning Xia of Shanghai Jiao Tong University, using the Shanghai area as an example, found that exposure to pesticides affects not only the reproductive health of women undergoing ART but also adversely affects their offspring. As more infants are born, some involving embryo culture and exposure to potentially inappropriate environmental factors, this could alter the phenotype of the offspring, such as Large Offspring Syndrome in cattle (257).

Like in cattle, ART can facilitate the development of human congenital overgrowth conditions, known as Beckwith-Wiedemann syndrome (BWS), which may later lead to molar pregnancies and embryonal tumor formation. BWS is an overgrowth and embryonal tumor susceptibility disorder linked to genetic or epigenetic abnormalities in the chromosome 11p15.5 region, causing abnormal expression of parental alleles. ART disrupts the DNA methylation of imprinting sites, supporting the notion that ART could lead to imprinting disorders, including BWS. Children conceived through ART are at a 4 to 10 times higher risk of developing BWS compared to those conceived naturally (258). Furthermore, long-term exposure to inappropriate environmental factors aligns with the “DOHaD theory” – that in addition to adult lifestyle and genetic inheritance, early life environmental factors, including nutrition, influence the risk of some non-communicable diseases in adulthood, such as obesity, diabetes, and cardiovascular diseases.

These studies indicate that the environmental and behavioral choices of mothers during pregnancy not only affect their own health but can also have profound effects on the health of their offspring. Therefore, understanding how maternal environmental factors influence the health of offspring through epigenetic mechanisms is of significant importance for the prevention and management of environment-related health issues.

#### 5.4.5 The application of epigenetics in reproductive medicine

The application of epigenetics in the field of reproductive medicine has significantly enhanced our understanding of the fertility process and provided new therapeutic strategies for treating infertility. According to a 2021 study published in “The Lancet,” the current global live birth rate for ART, such as *in vitro* fertilization (IVF), is below 30%. Epigenetics plays a crucial role in the development of embryos and the trophoblast layer. The use of ART during this critical period introduces a potential window of vulnerability where epigenetic changes can occur. This susceptibility is due to the significant epigenetic reprogramming that embryos undergo during early development, which can be influenced by the ART procedures. These procedures may include culture conditions, media composition, and handling techniques, all of which could potentially impact the epigenetic landscape of developing embryos, leading to lasting effects on gene expression and function. By regulating the methylation status of specific genes in ovarian cells, the success rate of IVF can be effectively improved (259), and the yield of high-quality embryos can be increased. This method increases the chances of fertilization and healthy pregnancy by optimizing the quality of oocytes and the early development of embryos.

Furthermore, research in epigenetics provides a solid scientific foundation for future reproductive health intervention strategies. These interventions could address the issue of declining fertility due to ovarian aging as age increases (260) and may play a role in treating reproductive endocrine diseases. For example, treatments for reproductive endocrine disorders like PCOS might find new directions from an epigenetic perspective.

In the realm of female reproductive health, studies in epigenetics have not only revealed the crucial roles of DNA methylation and histones post-translational modifications in ovarian function, oocyte maturation, and embryonic development but also emphasized the significant impact of environmental factors such as diet, lifestyle, and chemical exposures on gene expression. These impacts may extend to the pregnancy process and the long-term health of offspring.

On the other hand, epigenetic changes are reversible, and understanding the pathogenesis of epigenetic diseases could allow for therapeutic interventions by targeting these modifications. In-depth studies on epigenetics help elucidate the mechanisms of human reproduction and related diseases and offer potential therapeutic approaches. For example, abnormal DNA methylation plays a crucial role in the initiation and progression of endometrial cancer, leading to the silencing of Estrogen Receptor(ER) and Progesterone Receptor(PR) expression, increased genomic DNA instability, activation of oncogenes, and inactivation of tumor suppressor genes. Yanokura et al. (261) found that the abnormal hypermethylation of the CHFR mitotic checkpoint gene in endometrial cancer tumor cells is closely related to the sensitivity to taxane-based drugs, providing new intervention targets and guidance for tumor treatment.

As basic and clinical research advances, epigenetic regulation has been found to be significant in the pathogenesis, diagnosis, treatment, and prognosis assessment of various malignancies. Recent studies have shown that DNMT inhibitors, which competitively inhibit DNMT activity and block methylation reactions, have been effective in treating endometrial cancer in clinical settings (262). Clinical trials indicate that DNA demethylating agents can reverse platinum resistance in ovarian cancer patients, suggesting that epigenetic drug therapy has clinical benefits in treating chemotherapy-resistant or recurrent advanced ovarian cancer (263).

Exploring the epigenetic molecular regulatory mechanisms of human germ cell development not only provides a theoretical basis for inquiries into issues like epigenetic reprogramming of human germ cells, the establishment of pluripotency in early embryos, directed differentiation of stem cells into gametes, and transgenerational inheritance of DNA methylation but also holds significant implications for assessing the safety of assisted reproductive technologies, determining whether reproductive disorders will be inherited by offspring or across generations, researching recurrent miscarriages and embryonic arrest, and studying diseases related to abnormal development of reproductive cells in clinical settings.

In summary, research and applications of epigenetics in the field of reproductive medicine have provided new insights and therapeutic strategies, especially showing great potential in improving IVF and embryo quality.

## 6 Conclusion and future perspectives

Epigenetics plays a crucial role in women’s reproductive health, and environmental factors also have a potential impact on it by inducing epigenetic changes that affect female reproductive functions. External harmful environmental factors, such as PM2.5 and gaseous pollutants, can impact the female reproductive system, leading to infertility, pregnancy complications, and other issues. Additionally, harmful chemicals such as polycyclic aromatic hydrocarbons and the heavy metal cadmium can affect women’s reproductive health, potentially leading to preterm birth, miscarriage, and halted embryonic development among other adverse pregnancy outcomes. Long-term exposure to these environmental pollutants may lead to changes in the epigenetic markers of eggs and sperm, affecting the function of reproductive cells and thereby impacting the development of the embryo and fertility. Furthermore, environmental endocrine disruptors may affect epigenetic modifications, thereby interfering with the development of reproductive cells. Therefore, environmental factors and lifestyle choices are crucial for maintaining reproductive health.

The revelation of the impact of epigenetics and environmental factors on women’s reproductive health provides new approaches for treating infertility, pregnancy complications, and other diseases. By regulating the methylation status of specific genes in ovarian cells, the success rate of *in vitro* fertilization can be effectively increased and embryo quality improved. Meanwhile, studying how environmental factors influence reproductive health through epigenetic mechanisms is important for predicting and preventing developmental anomalies and improving offspring health.

Future research directions may include more in-depth studies on the role of epigenetics in women’s reproductive health and intervening in epigenetic changes through gene editing. Technologically, researchers might utilize epigenetics to address issues such as infertility and might also reduce the risk of embryos developing other diseases in adulthood by altering methylation in ovarian cells. For instance, the preconception period, pregnancy, and prebirth are becoming recognized as sensitive periods to the epigenetic impacts of environmental factors, which may increase the risk of chronic diseases in adulthood (including neurodegenerative diseases) (264). Additionally, future research will delve deeper into how environmental factors such as diet, lifestyle, and chemical exposure affect gene expression, and how these impacts, through epigenetic mechanisms, influence the health of offspring, thereby preventing and intervening in women’s reproductive health issues.

## References

[1] Zhang Y Cheng J Zhong C Xia Q Li Y Chen P. ESR1 regulates the obesity- and metabolism-differential gene MMAA to inhibit the occurrence and development of hepatocellular carcinoma. Front Oncol. (2022) 12:899969. doi: 10.3389/fonc.2022.899969 35795061 PMC9252523

[2] Vayeda M Ghanghar V Desai S Shah P Modi D Dave K. Improving menstrual hygiene management among adolescent girls in tribal areas of Gujarat: an evaluation of an implementation model integrating the government service delivery system. Sex Reprod Health Matters. (2021) 29:1992199. doi: 10.1080/26410397.2021.1992199 34939899 PMC8725704

[3] World Health Organization. Available online at: https://www.who.int/news-room/fact-sheets/detail/endometriosis (Accessed 27 September 2022).

[4] Mørch LS Skovlund CW Hannaford PC Iversen L Fielding S Lidegaard Ø. Contemporary hormonal contraception and the risk of breast cancer. N Engl J Med. (2017) 377:2228–39. doi: 10.1056/NEJMoa1700732 29211679

[5] Schairer C Lubin J Troisi R Sturgeon S Brinton L Hoover R. Menopausal estrogen and estrogen-progestin replacement therapy and breast cancer risk [published correction appears in JAMA 2000 Nov 22-29;284(20):2597. JAMA. (2000) 283:485–91. doi: 10.1001/jama.283.4.485 10659874

[6] Kaluscha S Domcke S Wirbelauer C Stadler MB Durdu S Burger L. Evidence that direct inhibition of transcription factor binding is the prevailing mode of gene and repeat repression by DNA methylation. Nat Genet. (2022) 54:1895–906. doi: 10.1038/s41588-022-01241-6 PMC9729108 36471082

[7] Okae H Toh H Sato T Hiura H Takahashi S Shirane K. Derivation of human trophoblast stem cells. Cell Stem Cell. (2018) 22:50–63. doi: 10.1016/j.stem.2017.11.004 29249463

[8] Tang L Zhang YY Liu WJ Fu Q Zhao J Liu Y. DNA methylation of promoter region inhibits Galectin-1 expression in BMSCs of aged mice. Am J Physiol Cell Physiol. (2024) 326(2):C429–C441. doi: 10.1152/ajpcell.00334.2023 38105757

[9] Zhong Z Xue Y Harris CJ Wang M Li Z Ke Y. MORC proteins regulate transcription factor binding by mediating chromatin compaction in active chromatin regions. Genome Biol. (2023) 24:96. doi: 10.1186/s13059-023-02939-4 37101218 PMC10131428

[10] Kamińska J Langa P Deptuła M Zieliński J Sachadyn P Wardowska A. Transcriptional activity of epigenetic remodeling genes declines in keratinocytes after in *vitro* expansion. Adv Med Sci. (2019) 64:274–9. doi: 10.1016/j.advms.2019.03.001 30901693

[11] Butz S Schmolka N Karemaker ID Villaseñor R Schwarz I Domcke S. DNA sequence and chromatin modifiers cooperate to confer epigenetic bistability at imprinting control regions. Nat Genet. (2022) 54:1702–10. doi: 10.1038/s41588-022-01210-z PMC9649441 36333500

[12] Luderer U Lim J Ortiz L Nguyen JD Shin JH Allen BD. Exposure to environmentally relevant concentrations of ambient fine particulate matter (PM2.5) depletes the ovarian follicle reserve and causes sex-dependent cardiovascular changes in apolipoprotein E null mice. Part Fibre Toxicol. (2022) 19:5. doi: 10.1186/s12989-021-00445-8 34996492 PMC8740366

[13] Zhou S Xi Y Chen Y Zhang Z Wu C Yan W. Ovarian dysfunction induced by chronic whole-body PM2.5 exposure. Small. (2020) 16:e2000845. doi: 10.1002/smll.202000845 32686359

[14] Wu S Hao G Zhang Y Chen X Ren H Fan Y. Poor ovarian response is associated with air pollutants: A multicentre study in China. EBioMedicine. (2022) 81:104084. doi: 10.1016/j.ebiom.2022.104084 35660784 PMC9163489

[15] LaPointe S Lee JC Nagy ZP Shapiro DB Chang HH Wang Y. Ambient traffic related air pollution in relation to ovarian reserve and oocyte quality in young, healthy oocyte donors. Environ Int. (2024) 183:108382. doi: 10.1016/j.envint.2023.108382 38103346 PMC10871039

[16] Dos Anjos LG de Almeida BC Baracat EC Al-Hendy A Yang Q Carvalho KC. Gene expression profile of uterine leiomyoma from women exposed to different air pollution levels in metropolitan cities of sao paulo, Brazil. Int J Mol Sci. (2023) 24:2431. doi: 10.3390/ijms24032431 36768749 PMC9917088

[17] Goin DE Sudat S Riddell C Morello-Frosch R Apte JS Glymour MM. Hyperlocalized measures of air pollution and preeclampsia in oakland, california. Environ Sci Technol. (2021) 55:14710–9. doi: 10.1021/acs.est.1c02151 PMC8968652 34648281

[18] Juan-Reyes SS Gómez-Oliván LM Juan-Reyes NS Islas-Flores H Dublán-García O Orozco-Hernández JM. Women with preeclampsia exposed to air pollution during pregnancy: Relationship between oxidative stress and neonatal disease - Pilot study. Sci Total Environ. (2023) 871:161858. doi: 10.1016/j.scitotenv.2023.161858 36716872

[19] Song W Li A Sha QQ Liu SY Zhou Y Zhou CY. Maternal exposure to 4-vinylcyclohexene diepoxide during pregnancy induces subfertility and birth defects of offspring in mice. Sci Total Environ. (2023) 859:160431. doi: 10.1016/j.scitotenv.2022.160431 36423845

[20] Dai M Huang W Huang X Ma C Wang R Tian P. BPDE, the migration and invasion of human trophoblast cells, and occurrence of miscarriage in humans: roles of a novel lncRNA-HZ09. Environ Health Perspect. (2023) 131:17009. doi: 10.1289/EHP10477 36719213 PMC9888265

[21] ssah I Duah MS Arko-Mensah J Bawua SA Agyekum TP Fobil JN. Exposure to metal mixtures and adverse pregnancy and birth outcomes: A systematic review. Sci Total Environ. (2024) 908:168380. doi: 10.1016/j.scitotenv.2023.168380 37963536

[22] Yano S Ishiuchi T Abe S Namekawa SH Huang G Ogawa Y. Histone H3K36me2 and H3K36me3 form a chromatin platform essential for DNMT3A-dependent DNA methylation in mouse oocytes. Nat Commun. (2022) 13:4440. doi: 10.1038/s41467-022-32141-2 35922445 PMC9349174

[23] Malachowski T Chandradoss KR Boya R Zhou L Cook AL Su C. Spatially coordinated heterochromatinization of long synaptic genes in fragile X syndrome. Cell. (2023) 186:5840–58. doi: 10.1016/j.cell.2023.11.019 PMC10794044 38134876

[24] Singh K Rustagi Y Abouhashem AS Tabasum S Verma P Hernandez E. Genome-wide DNA hypermethylation opposes healing in patients with chronic wounds by impairing epithelial-mesenchymal transition. J Clin Invest. (2022) 132:e157279. doi: 10.1172/JCI157279 35819852 PMC9433101

[25] He L Huang H Bradai M Zhao C You Y Ma J. DNA methylation-free Arabidopsis reveals crucial roles of DNA methylation in regulating gene expression and development. Nat Commun. (2022) 13:1335. doi: 10.1038/s41467-022-28940-2 35288562 PMC8921224

[26] Xuan Lin QX Sian S An O Thieffry D Jha S Benoukraf T. MethMotif: an integrative cell specific database of transcription factor binding motifs coupled with DNA methylation profiles. Nucleic Acids Res. (2019) 47:D145–54. doi: 10.1093/nar/gky1005 PMC6323897 30380113

[27] Chen Z Zhang Y. Role of mammalian DNA methyltransferases in development. Annu Rev Biochem. (2020) 89:135–58. doi: 10.1146/annurev-biochem-103019-102815 31815535

[28] Li Y Zhang Z Chen J Liu W Lai W Liu B. Stella safeguards the oocyte methylome by preventing *de novo* methylation mediated by DNMT1. Nature. (2018) 564:136–40. doi: 10.1038/s41586-018-0751-5 30487604

[29] Andrews S Krueger C Mellado-Lopez M Hemberger M Dean W Perez-Garcia V. Mechanisms and function of *de novo* DNA methylation in placental development reveals an essential role for DNMT3B. Nat Commun. (2023) 14:371. doi: 10.1038/s41467-023-36019-9 36690623 PMC9870994

[30] Dura M Teissandier A Armand M Barau J Lapoujade C Fouchet P. DNMT3A-dependent DNA methylation is required for spermatogonial stem cells to commit to spermatogenesis. Nat Genet. (2022) 54:469–80. doi: 10.1038/s41588-022-01040-z 35410378

[31] Noh KM Wang H Kim HR Wenderski W Fang F Li CH. Engineering of a histone-recognition domain in dnmt3a alters the epigenetic landscape and phenotypic features of mouse ESCs. ” Mol Cell. (2015) 59,1:89–103. doi: 10.1016/j.molcel.2015.05.017 26073541 PMC4491196

[32] He W Zhang X Zhang Y Zheng W Xiong Z Hu X. Reduced self-diploidization and improved survival of semi-cloned mice produced from androgenetic haploid embryonic stem cells through overexpression of dnmt3b. Stem Cell Rep. (2018) 10:477–93. doi: 10.1016/j.stemcr.2017.12.024 PMC5831042 29396184

[33] Weinberg DN Papillon-Cavanagh S Chen H Yue Y Chen X Rajagopalan KN. The histone mark H3K36me2 recruits DNMT3A and shapes the intergenic DNA methylation landscape. ” Nat. (2019) 573:281–6. doi: 10.1038/s41586-019-1534-3 PMC6742567 31485078

[34] Kenjiro S Shirane K Miura F Ito T Lorincz MC. NSD1-deposited H3K36me2 directs *de novo* methylation in the mouse male germline and counteracts Polycomb-associated silencing. ” Nat Genet. (2020) 52:1088–98. doi: 10.1038/s41588-020-0689-z 32929285

[35] Gu T Hao D Woo J Huang TW Guo L Lin X. The disordered N-terminal domain of DNMT3A recognizes H2AK119ub and is required for postnatal development. Nat Genet. (2022) 54:625–36. doi: 10.1038/s41588-022-01063-6 PMC9295050 35534561

[36] Qin L Qiao C Sheen V Wang Y Lu J. DNMT3L promotes neural differentiation by enhancing STAT1 and STAT3 phosphorylation independent of DNA methylation. Prog Neurobiol. (2021) 201:102028. doi: 10.1016/j.pneurobio.2021.102028 33636226

[37] Behluli L Fontanilla AM Andessner-Angleitner L Tolar N Molina JM Gahurova L. Expression analysis suggests that DNMT3L is required for oocyte *de novo* DNA methylation only in Muridae and Cricetidae rodents. Epigenet Chromatin. (2023) 16:43. doi: 10.1186/s13072-023-00518-2 PMC10625200 37924163

[38] Finnegan AI Kim S Jin H Gapinske M Woods WS Perez-Pinera P. Epigenetic engineering of yeast reveals dynamic molecular adaptation to methylation stress and genetic modulators of specific DNMT3 family members. Nucleic Acids Res. (2020) 48:4081–99. doi: 10.1093/nar/gkaa161 PMC7192628 32187373

[39] Wang Q Liang N Yang T Li Y Li J Huang Q. DNMT1-mediated methylation of BEX1 regulates stemness and tumorigenicity in liver cancer. J Hepatol. (2021) 75:1142–53. doi: 10.1016/j.jhep.2021.06.025 34217777

[40] Hahm JY Park JW Kang JY Park J Kim CH Kim JY. Acetylation of UHRF1 regulates hemi-methylated DNA binding and maintenance of genome-wide DNA methylation. Cell Rep. (2020) 32:107958. doi: 10.1016/j.celrep.2020.107958 32726623

[41] Mattei AL Bailly N Meissner A. DNA methylation: a historical perspective. Trends Genet. (2022) 38:676–707. doi: 10.1016/j.tig.2022.03.010 35504755

[42] Schneider M Trummer C Stengl A Zhang P Szwagierczak A Cardoso MC. Systematic analysis of the binding behaviour of UHRF1 towards different methyl- and carboxylcytosine modification patterns at CpG dyads. PloS One. (2020) 15:e0229144. doi: 10.1371/journal.pone.0229144 32084194 PMC7034832

[43] Fang J Cheng J Wang J Zhang Q Liu M Gong R. Hemi-methylated DNA opens a closed conformation of UHRF1 to facilitate its histone recognition. Nat Commun. (2016) 7:11197. doi: 10.1038/ncomms11197 27045799 PMC4822050

[44] Tahiliani M Koh KP Shen Y Pastor WA Bandukwala H Brudno Y. Conversion of 5-methylcytosine to 5-hydroxymethylcytosine in mammalian DNA by MLL partner TET1. Sci (New York N.Y.). (2009) 324:930–5:5929. doi: 10.1126/science.1170116 PMC2715015 19372391

[45] Kriaucionis S Heintz N. The nuclear DNA base 5-hydroxymethylcytosine is present in Purkinje neurons and the brain. Science. (2009) 324:929–30. doi: 10.1126/science.1169786 PMC3263819 19372393

[46] Bhutani N Burns DM Blau HM. DNA demethylation dynamics. Cell. (2011) 146:866–72. doi: 10.1016/j.cell.2011.08.042 PMC3236603 21925312

[47] Huang Y Li L An G Yang X Cui M Song X. Single-cell multi-omics sequencing of human spermatogenesis reveals a DNA demethylation event associated with male meiotic recombination. Nat Cell Biol. (2023) 25:1520–34. doi: 10.1038/s41556-023-01232-7 37723297

[48] Ghoneim M Fuchs HA Musselman CA. Histone tail conformations: a fuzzy affair with DNA. Trends Biochem Sci. (2021) 46:564–78. doi: 10.1016/j.tibs.2020.12.012 PMC8195839 33551235

[49] Luger K Mäder AW Richmond RK Sargent DF Richmond TJ. Crystal structure of the nucleosome core particle at 2. 8 A resolution. Nat. (1997) 389:251–60. doi: 10.1038/38444 9305837

[50] Li S Peng Y Landsman D Panchenko AR. DNA methylation cues in nucleosome geometry, stability and unwrapping. Nucleic Acids Res. (2022) 50:1864–74. doi: 10.1093/nar/gkac097 PMC8881801 35166834

[51] Peng Y Li S Landsman D Panchenko AR. Histone tails as signaling antennas of chromatin. Curr Opin Struct Biol. (2021) 67:153–60. doi: 10.1016/j.sbi.2020.10.018 PMC8096652 33279866

[52] Gao G Hausmann S Flores NM Benitez AM Shen J Yang X. The NFIB/CARM1 partnership is a driver in preclinical models of small cell lung cancer. Nat Commun. (2023) 14:363. doi: 10.1038/s41467-023-35864-y 36690626 PMC9870865

[53] Gao Y Sheng X Tan D Kim S Choi S Paudel S. Identification of histone lysine acetoacetylation as a dynamic post-translational modification regulated by HBO1. Adv Sci (Weinh). (2023) 10:e2300032. doi: 10.1002/advs.202300032 37382194 PMC10477889

[54] Liao L He Y Li SJ Yu XM Liu ZC Liang YY. Lysine 2-hydroxyisobutyrylation of NAT10 promotes cancer metastasis in an ac4C-dependent manner. Cell Res. (2023) 33:355–71. doi: 10.1038/s41422-023-00793-4 PMC10156899 36882514

[55] Hu Z Wei F Su Y Wang Y Shen Y Fang Y. Histone deacetylase inhibitors promote breast cancer metastasis by elevating NEDD9 expression. Signal Transduct Target Ther. (2023) 8:11. doi: 10.1038/s41392-022-01221-6 36604412 PMC9816171

[56] Wang ZA Cole PA. The chemical biology of reversible lysine post-translational modifications. Cell Chem Biol. (2020) 27:953–69. doi: 10.1016/j.chembiol.2020.07.002 PMC7487139 32698016

[57] Jambhekar A Dhall A Shi Y. Roles and regulation of histone methylation in animal development. Nat Rev Mol Cell Biol. (2019) 20:625–41. doi: 10.1038/s41580-019-0151-1 PMC6774358 31267065

[58] Haws SA Yu D Ye C Wille CK Nguyen LC Krautkramer KA. Methyl-metabolite depletion elicits adaptive responses to support heterochromatin stability and epigenetic persistence. Mol Cell. (2020) 78:210–23. doi: 10.1016/j.molcel.2020.03.004 PMC7191556 32208170

[59] Yi Y Ge S. Targeting the histone H3 lysine 79 methyltransferase DOT1L in MLL-rearranged leukemias. J Hematol Oncol. (2022) 15:35. doi: 10.1186/s13045-022-01251-1 35331314 PMC8944089

[60] Qiu J Xu B Ye D Ren D Wang S Benci JL. Cancer cells resistant to immune checkpoint blockade acquire interferon-associated epigenetic memory to sustain T cell dysfunction. Nat Cancer. (2023) 4:43–61. doi: 10.1038/s43018-022-00490-y 36646856

[61] Padeken J Methot SP Gasser SM. Establishment of H3K9-methylated heterochromatin and its functions in tissue differentiation and maintenance. Nat Rev Mol Cell Biol. (2022) 23:623–40. doi: 10.1038/s41580-022-00483-w PMC9099300 35562425

[62] Li Z Duan S Hua X Xu X Li Y Menolfi D. Asymmetric distribution of parental H3K9me3 in S phase silences L1 elements. Nature. (2023) 623:643–51. doi: 10.1038/s41586-023-06711-3 PMC11034792 37938774

[63] Husmann D Gozani O. Histone lysine methyltransferases in biology and disease. Nat Struct Mol Biol. (2019) 26:880–9. doi: 10.1038/s41594-019-0298-7 PMC6951022 31582846

[64] Greer EL Shi Y. Histone methylation: a dynamic mark in health, disease and inheritance. Nat Rev Genet. (2012) 13:343–57. doi: 10.1038/nrg3173 PMC4073795 22473383

[65] Xiao Y Zhao C Tai Y Li B Lan T Lai E. STING mediates hepatocyte pyroptosis in liver fibrosis by Epigenetically activating the NLRP3 inflammasome. Redox Biol. (2023) 62:102691. doi: 10.1016/j.redox.2023.102691 37018971 PMC10106968

[66] Gray ZH Chakraborty D Duttweiler RR Alekbaeva GD Murphy SE Chetal K. Epigenetic balance ensures mechanistic control of MLL amplification and rearrangement. Cell. (2023) 186:4528–4545.e18. doi: 10.1016/j.cell.2023.09.009 37788669 PMC10591855

[67] Huang X Zhang X Zong L Gao Q Zhang C Wei R. Gene body methylation safeguards ribosomal DNA transcription by preventing PHF6-mediated enrichment of repressive histone mark H4K20me3. J Biol Chem. (2021) 297:101195. doi: 10.1016/j.jbc.2021.101195 34520760 PMC8511956

[68] Jiang Q Stachelscheid J Bloehdorn J Pacholewska A Aszyk C Grotenhuijs F. Oncogenic role and target properties of the lysine-specific demethylase KDM1A in chronic lymphocytic leukemia. Blood. (2023) 142:44–61. doi: 10.1182/blood.2022017230 37023372

[69] Zhang J Wang H Chen H Li H Xu P Liu B. ATF3 -activated accelerating effect of LINC00941/lncIAPF on fibroblast-to-myofibroblast differentiation by blocking autophagy depending on ELAVL1/HuR in pulmonary fibrosis. Autophagy. (2022) 18:2636–55. doi: 10.1080/15548627.2022.2046448 PMC9629064 35427207

[70] Xie S Jiang C Wu M Ye Y Wu B Sun X. Dietary ketone body-escalated histone acetylation in megakaryocytes alleviates chemotherapy-induced thrombocytopenia. Sci Transl Med. (2022) 14:eabn9061. doi: 10.1126/scitranslmed.abn9061 36449600

[71] Tessarz P Kouzarides T. Histone core modifications regulating nucleosome structure and dynamics. Nat Rev Mol Cell Biol. (2014) 15:703–8. doi: 10.1038/nrm3890 25315270

[72] Kim TH Nosella ML Bolik-Coulon N Harkness RW Huang SK Kay LE. Correlating histone acetylation with nucleosome core particle dynamics and function. Proc Natl Acad Sci U S A. (2023) 120:e2301063120. doi: 10.1073/pnas.2301063120 37011222 PMC10104578

[73] Voss AK Thomas T. Histone lysine and genomic targets of histone acetyltransferases in mammals. Bioessays. (2018) 40:e1800078. doi: 10.1002/bies.201800078 30144132

[74] von Knethen A Brüne B. Histone deacetylation inhibitors as therapy concept in sepsis. Int J Mol Sci. (2019) 20:346. doi: 10.3390/ijms20020346 30654448 PMC6359123

[75] Li H Chen X Xu J Zhu L Li C Sun X. GRP/GRPR enhances alcohol-associated liver injury through the IRF1-mediated Caspase-1 inflammasome and NOX2-dependent ROS pathway. Hepatology. (2024) 79:392–408. doi: 10.1097/HEP.0000000000000531 37409771

[76] Cai L Sutter BM Li B Tu BP. Acetyl-CoA induces cell growth and proliferation by promoting the acetylation of histones at growth genes. Mol Cell. (2011) 42:426–37. doi: 10.1016/j.molcel.2011.05.004 PMC3109073 21596309

[77] Wellen KE Hatzivassiliou G Sachdeva UM Bui TV Cross JR Thompson CB. ATP-citrate lyase links cellular metabolism to histone acetylation. Science. (2009) 324:1076–80. doi: 10.1126/science.1164097 PMC2746744 19461003

[78] Izzo LT Trefely S Demetriadou C Drummond JM Mizukami T Kuprasertkul N. Acetylcarnitine shuttling links mitochondrial metabolism to histone acetylation and lipogenesis. Sci Adv. (2023) 9:eadf0115. doi: 10.1126/sciadv.adf0115 37134161 PMC10156126

[79] Morrow MR Batchuluun B Wu J Ahmadi E Leroux JM Mohammadi-Shemirani P. Inhibition of ATP-citrate lyase improves NASH, liver fibrosis, and dyslipidemia. Cell Metab. (2022) 34:919–936.e8. doi: 10.1016/j.cmet.2022.05.004 35675800

[80] Mao Y Zhang J Zhou Q He X Zheng Z Wei Y. Hypoxia induces mitochondrial protein lactylation to limit oxidative phosphorylation. Cell Res. (2024) 34:13–30. doi: 10.1038/s41422-023-00864-6 38163844 PMC10770133

[81] Mews P Donahue G Drake AM Luczak V Abel T Berger SL. Acetyl-CoA synthetase regulates histone acetylation and hippocampal memory. Nature. (2017) 546:381–6. doi: 10.1038/nature22405 PMC5505514 28562591

[82] Mews P Egervari G Nativio R Sidoli S Donahue G Lombroso SI. Alcohol metabolism contributes to brain histone acetylation. Nature. (2019) 574:717–21. doi: 10.1038/s41586-019-1700-7 PMC6907081 31645761

[83] Zhang D Tang Z Huang H Zhou G Cui C Weng Y. Metabolic regulation of gene expression by histone lactylation. Nature. (2019) 574:575–80. doi: 10.1038/s41586-019-1678-1 PMC6818755 31645732

[84] Wan N Wang N Yu S Zhang H Tang S Wang D. Cyclic immonium ion of lactyllysine reveals widespread lactylation in the human proteome. Nat Methods. (2022) 19:854–64. doi: 10.1038/s41592-022-01523-1 35761067

[85] Villanueva L Alvarez-Errico D Esteller M. The contribution of epigenetics to cancer immunotherapy. Trends Immunol. (2020) 41:676–91. doi: 10.1016/j.it.2020.06.002 32622854

[86] Slack FJ Chinnaiyan AM. The role of non-coding RNAs in oncology. Cell. (2019) 179:1033–55. doi: 10.1016/j.cell.2019.10.017 PMC7347159 31730848

[87] An Y Duan H. The role of m6A RNA methylation in cancer metabolism. Mol Cancer. (2022) 21:14. doi: 10.1186/s12943-022-01500-4 35022030 PMC8753874

[88] Raju GSR Pavitra E Bandaru SS Varaprasad GL Nagaraju GP Malla RR. HOTAIR: a potential metastatic, drug-resistant and prognostic regulator of breast cancer. Mol Cancer. (2023) 22:65. doi: 10.1186/s12943-023-01765-3 36997931 PMC10061914

[89] Xue C Li G Zheng Q Gu X Bao Z Lu J. The functional roles of the circRNA/Wnt axis in cancer. Mol Cancer. (2022) 21:108. doi: 10.1186/s12943-022-01582-0 35513849 PMC9074313

[90] van Zonneveld AJ Zhao Q Rotmans JI Bijkerk R. Circulating non-coding RNAs in chronic kidney disease and its complications. Nat Rev Nephrol. (2023) 19:573–86. doi: 10.1038/s41581-023-00725-w 37286733

[91] Welsh SA Gardini A. Genomic regulation of transcription and RNA processing by the multitasking Integrator complex. Nat Rev Mol Cell Biol. (2023) 24:204–20. doi: 10.1038/s41580-022-00534-2 PMC9974566 36180603

[92] Cornes E Bourdon L Singh M Mueller F Quarato P Wernersson E. piRNAs initiate transcriptional silencing of spermatogenic genes during C. elegans germline development. Dev Cell. (2022) 57:180–196.e7. doi: 10.1016/j.devcel.2021.11.025 34921763 PMC8796119

[93] Ruggieri F Jonas K Ferracin M Dengler M Jäger V Pichler M. MicroRNAs as regulators of tumor metabolism. Endocr Relat Cancer. (2023) 30:e220267. doi: 10.1530/ERC-22-0267 37224081

[94] Lin C Ma M Zhang Y Li L Long F Xie C. The N6-methyladenosine modification of circALG1 promotes the metastasis of colorectal cancer mediated by the miR-342-5p/PGF signalling pathway [published correction appears in Mol Cancer. Mol Cancer. (2022) 21:80. doi: 10.1186/s12943-022-01560-6 35305647 PMC8933979

[95] Liu Y Li C Fang L Wang L Liu H Tian H. Lipid metabolism-related lncRNA SLC25A21-AS1 promotes the progression of oesophageal squamous cell carcinoma by regulating the NPM1/c-Myc axis and SLC25A21 expression. Clin Transl Med. (2022) 12:e944. doi: 10.1002/ctm2.944 35735113 PMC9218933

[96] Luo Y Huang S Wei J Zhou H Wang W Yang J. Long noncoding RNA LINC01606 protects colon cancer cells from ferroptotic cell death and promotes stemness by SCD1-Wnt/β-catenin-TFE3 feedback loop signalling. Clin Transl Med. (2022) 12:e752. doi: 10.1002/ctm2.752 35485210 PMC9052012

[97] Zhang N Zhang X Xu W Zhang X Mu Z. CircRNA_103948 inhibits autophagy in colorectal cancer in a ceRNA manner. Ann N Y. Acad Sci. (2021) 1503:88–101. doi: 10.1111/nyas.14679 34480353

[98] Xi Y Shen Y Wu D Zhang J Lin C Wang L. CircBCAR3 accelerates esophageal cancer tumorigenesis and metastasis via sponging miR-27a-3p. Mol Cancer. (2022) 21:145. doi: 10.1186/s12943-022-01615-8 35840974 PMC9284725

[99] Hattori N Liu YY Ushijima T. DNA methylation analysis. Methods Mol Biol. (2023) 2691:165–83. doi: 10.1007/978-1-0716-3331-1_13 37355545

[100] Ma F Jiang S Zhang CY. Recent advances in histone modification and histone modifying enzyme assays. Expert Rev Mol Diagn. (2019) 19:27–36. doi: 10.1080/14737159.2019.1559053 30563379

[101] Salmen F De Jonghe J Kaminski TS Alemany A Parada GE Verity-Legg J. High-throughput total RNA sequencing in single cells using VASA-seq. Nat Biotechnol. (2022) 40:1780–93. doi: 10.1038/s41587-022-01361-8 PMC9750877 35760914

[102] Rauscher GH Kresovich JK Poulin M Yan L Macias V Mahmoud AM. Exploring DNA methylation changes in promoter, intragenic, and intergenic regions as early and late events in breast cancer formation. BMC Cancer. (2015) 15:816. doi: 10.1186/s12885-015-1777-9 26510686 PMC4625569

[103] Zou LS Erdos MR Taylor DL Chines PS Varshney A McDonnell Genome Institute. BoostMe accurately predicts DNA methylation values in whole-genome bisulfite sequencing of multiple human tissues. BMC Genomics. (2018) 19:390. doi: 10.1186/s12864-018-4766-y 29792182 PMC5966887

[104] Weber M Davies JJ Wittig D Oakeley EJ Haase M Lam WL. Chromosome-wide and promoter-specific analyses identify sites of differential DNA methylation in normal and transformed human cells. Nat Genet. (2005) 37:853–62. doi: 10.1038/ng1598 16007088

[105] Jacinto FV Ballestar E Esteller M. Methyl-DNA immunoprecipitation (MeDIP): hunting down the DNA methylome. Biotechniques. (2008) 44:35, 37, 39. doi: 10.2144/000112708 18254377

[106] Bibikova M Barnes B Tsan C Ho V Klotzle B Le JM. High density DNA methylation array with single CpG site resolution. Genomics. (2011) 98:288–95. doi: 10.1016/j.ygeno.2011.07.007 21839163

[107] Herman JG Graff JR Myöhänen S Nelkin BD Baylin SB. Methylation-specific PCR: a novel PCR assay for methylation status of CpG islands. Proc Natl Acad Sci U S A. (1996) 93:9821–6. doi: 10.1073/pnas.93.18.9821 PMC38513 8790415

[108] Masser DR Berg AS Freeman WM. Focused, high accuracy 5-methylcytosine quantitation with base resolution by benchtop next-generation sequencing. Epigenet Chromatin. (2013) 6:33. doi: 10.1186/1756-8935-6-33 PMC3907040 24279302

[109] Meissner A Gnirke A Bell GW Ramsahoye B Lander ES Jaenisch R. Reduced representation bisulfite sequencing for comparative high-resolution DNA methylation analysis. Nucleic Acids Res. (2005) 33:5868–77. doi: 10.1093/nar/gki901 PMC1258174 16224102

[110] Javadmanesh A Mojtabanezhad Shariatpanahi A Shams Davodly E Azghandi M Yassi M Heidari M. MS-HRM protocol: a simple and low-cost approach for technical validation of next-generation methylation sequencing data. Mol Genet Genomics. (2022) 297:1101–9. doi: 10.1007/s00438-022-01906-1 35616708

[111] Nakato R Sakata T. Methods for ChIP-seq analysis: A practical workflow and advanced applications. Methods. (2021) 187:44–53. doi: 10.1016/j.ymeth.2020.03.005 32240773

[112] Teng M. Statistical analysis in chIP-seq-related applications. Methods Mol Biol. (2023) 2629:169–81. doi: 10.1007/978-1-0716-2986-4_9 36929078

[113] Kaya-Okur HS Wu SJ Codomo CA Pledger ES Bryson TD Henikoff JG. CUT&Tag for efficient epigenomic profiling of small samples and single cells. Nat Commun. (2019) 10:1930. doi: 10.1038/s41467-019-09982-5 31036827 PMC6488672

[114] Sidoli S Kori Y Lopes M Yuan Z-F Kim HJ Kulej K. One minute analysis of 200 histone posttranslational modifications by direct injection mass spectrometry. Genome Res. (2019) 29:978–87. doi: 10.1101/gr.247353.118 PMC6581051 31123082

[115] Brasil PE De Castro L Hasslocher-Moreno AM Sangenis LH Braga JU. ELISA versus PCR for diagnosis of chronic Chagas disease: systematic review and meta-analysis. BMC Infect Dis. (2010) 10:337. doi: 10.1186/1471-2334-10-337 21108793 PMC3004908

[116] Kauter J Damek F Schares G Blaga R Schott F Deplazes P. Detection of Toxoplasma gondii-specific antibodies in pigs using an oral fluid-based commercial ELISA: Advantages and limitations. Int J Parasitol. (2023) 53:523–30. doi: 10.1016/j.ijpara.2022.11.003 36587725

[117] Kashina AS Yates Iii JR. Analysis of arginylated peptides by subtractive edman degradation. Methods Mol Biol. (2023) 2620:153–5. doi: 10.1007/978-1-0716-2942-0_20 37010761

[118] Ma P Amemiya HM He LL Gandhi SJ Nicol R Bhattacharyya RP. Bacterial droplet-based single-cell RNA-seq reveals antibiotic-associated heterogeneous cellular states. Cell. (2023) 186:877–891.e14. doi: 10.1016/j.cell.2023.01.002 36708705 PMC10014032

[119] Cao M Zhao J Hu G. Genome-wide methods for investigating long noncoding RNAs. Biomedicine pharmacotherapy = Biomedecine pharmacotherapie. (2019) 111:395–401. doi: 10.1016/j.biopha.2018.12.078 30594777 PMC6401243

[120] Taylor SC Posch A. The design of a quantitative western blot experiment. BioMed Res Int. (2014) 2014:361590. doi: 10.1155/2014/361590 24738055 PMC3971489

[121] Zhao J Ohsumi TK Kung JT Ogawa Y Grau DJ Sarma K. Genome-wide identification of polycomb-associated RNAs by RIP-seq. Mol Cell. (2010) 40:939–53. doi: 10.1016/j.molcel.2010.12.011 PMC3021903 21172659

[122] Jing Y Lin S Wang FT Yu JW Jiang F. [Detection of Gene Abnormalities in 43 Cases of Chronic Lymphocytic Leukemia by Fluorescence in Situ Hybridization]. Zhongguo Shi Yan Xue Ye Xue Za Zhi. (2018) 26(4):1038–43. doi: 10.7534/j.issn.1009-2137.2018.04.016. 30111404

[123] Huang J Ju Z Li Q Hou Q Wang C Li J. Solexa sequencing of novel and differentially expressed microRNAs in testicular and ovarian tissues in Holstein cattle. Int J Biol Sci. (2011) 7:1016–26. doi: 10.7150/ijbs.7.1016 PMC3164151 21912509

[124] Ghosh K Chatterjee B Behera P Kanade SR. The carcinogen cadmium elevates CpG-demethylation and enrichment of NFYA and E2F1 in the promoter of oncogenic PRMT5 and EZH2 methyltransferases resulting in their elevated expression in vitro. Chemosphere. (2020) 242:125186. doi: 10.1016/j.chemosphere.2019.125186 31675590

[125] Aigner GP Pittl V Fiechtner B Egger B Šrut M Höckner M. Common mechanisms cannot explain time- and dose-dependent DNA methylation changes in earthworms exposed to cadmium. Sci Total Environ. (2022) 812:151468. doi: 10.1016/j.scitotenv.2021.151468 34742794

[126] Sun L Mu Y Xu L Han X Gu W Zhang M. Transgenerational inheritance of wing development defects in Drosophila melanogaster induced by cadmium. Ecotoxicol Environ Saf. (2023) 250:114486. doi: 10.1016/j.ecoenv.2022.114486 36587412

[127] Chen M Li X Fan R Yang J Jin X Hamid S. Cadmium induces BNIP3-dependent autophagy in chicken spleen by modulating miR-33-AMPK axis. Chemosphere. (2018) 194:396–402. doi: 10.1016/j.chemosphere.2017.12.026 29223809

[128] Sharara FI Seifer DB Flaws JA. Environmental toxicants and female reproduction. Fertil Steril. (1998) 70:613–22. doi: 10.1016/S0015-0282(98)00253-2 9797086

[129] Baranski B Szymczyk I. Effects of mercury vapors upon reproductive function of the female white rat. Medycyna Pracy. (1973) 24:249–61.

[130] Davis BJ Price HC O'Connor RW Fernando R Rowland AS Morgan DL. Mercury vapor and female reproductive toxicity. Toxicol Sci. (2001) 59:291–6. doi: 10.1093/toxsci/59.2.291 11158722

[131] Lamperti AA Printz RH. Effects of mercuric chloride on the reproductive cycle of the female hamster. Biol Reprod. (1973) 8:378–87. doi: 10.1093/biolreprod/8.3.378 4735707

[132] Dansereau M Larivière DN Du Tremblay D Belanger D. Reproductive performance of two generations of female semidomesticated mink fed diets containing organic mercury contaminated freshwater fish. Arch Environ Contam Toxicol. (1999) 36:221–6. doi: 10.1007/s002449900464 9888969

[133] Haugen BR. Drugs that suppress TSH or cause central hypothyroidism. Best Pract Res Clin Endocrinol Metab. (2009) 23(6):793–800. doi: 10.1016/j.beem.2009.08.003 PMC2784889 19942154

[134] Gonsioroski A Mourikes VE Flaws JA. Endocrine Disruptors in Water and Their Effects on the Reproductive System. Int J Mol Sci. (2020) 21(6):1929. doi: 10.3390/ijms21061929 32178293 PMC7139484

[135] Rattan S Flaws JA. The epigenetic impacts of endocrine disruptors on female reproduction across generations. Biol Reprod. (2019) 101:635–44. doi: 10.1093/biolre/ioz081 PMC6791056 31077281

[136] Signorile PG Spugnini EP Citro G Viceconte R Vincenzi B Baldi F. Endocrine disruptors in *utero* cause ovarian damages linked to endometriosis. Front Biosci -Elite. (2012) 4:1724–30. doi: 10.2741/e493 22201988

[137] WHO. State of the science of endocrine disrupting chemicals 2012. In: Summary for decision-makers. WHO, Geneva, Switzerland (2012).

[138] Moreira Fernandez MA Cardeal ZL Carneiro MM André LC. Study of possible association between endometriosis and phthalate and bisphenol A by biomarkers analysis. J Pharm Biomed Anal. (2019) 172:238–42. doi: 10.1016/j.jpba.2019.04.048 31063881

[139] Ji C Song Q Chen Y Zhou Z Wang P Liu J. The potential endocrine disruption of pesticide transformation products (TPs): The blind spot of pesticide risk assessment. Environ Int. (2020) 137:105490. doi: 10.1016/j.envint.2020.105490 32007685

[140] Kaushal J Khatri M Arya SK. A treatise on Organophosphate pesticide pollution: Current strategies and advancements in their environmental degradation and elimination. Ecotoxicol. Environ Saf. (2021) 207:111483. doi: 10.1016/j.ecoenv.2020.111483 33120277

[141] Mahmood I Imadi SR Shazadi K Gul A Hakeem KR. Plant, soil and microbes. In: Effects of pesticides on environment. Springer, Berlin/Heidelberg, Germany (2016). p. 253–69.

[142] Anand N Chakraborty P Ray S. Human exposure to organochlorine, pyrethroid and neonicotinoid pesticides: Comparison between urban and semi-urban regions of India. Environ pollut. (2021) 270:116156. doi: 10.1016/j.envpol.2020.116156 33321437

[143] Jugan J Lind PM Salihovic S Stubleski J Karrman A Lind L. The associations between p,p′-DDE levels and plasma levels of lipoproteins and their subclasses in an elderly population determined by analysis of lipoprotein content. Lipids Health Dis. (2020) 19:249. doi: 10.1186/s12944-020-01417-1 33287856 PMC7722417

[144] Yaglova N Tsomartova D Yaglov V. Differences in production of adrenal steroid hormones in pubertal rats exposed to low doses of the endocrine disruptor DDT during prenatal and postnatal development. Biochem (Mosc.) Suppl. Ser B Biomed Chem. (2018) 12:80–6. doi: 10.1134/S1990750818010122

[145] Wang L Qie Y Yang Y Zhao Q. Binding and activation of estrogen-related receptor γ: A novel molecular mechanism for the estrogenic disruption effects of DDT and its metabolites. Environ Sci Technol. (2022) 56:12358–67. doi: 10.1021/acs.est.1c08624 35947429

[146] Yaglova NV Tsomartova DA Obernikhin SS Yaglov VV Nazimova SV Tsomartova ES. Differential disrupting effects of prolonged low-dose exposure to dichlorodiphenyltrichloroethane on androgen and estrogen production in males. Int J Mol Sci. (2021) 22:3155. doi: 10.3390/ijms22063155 33808818 PMC8003643

[147] Lai KP Tim Leung CC Boncan DAT Tam N Lin X Wang SY. Hypoxia-induced epigenetic transgenerational miRNAs dysregulation involved in reproductive impairment of ovary. Chem Biol Interact. (2022) 367:110176. doi: 10.1016/j.cbi.2022.110176 36096162

[148] González-Rojo S Lombó M Fernández-Díez C Herráez MP. Male exposure to bisphenol a impairs spermatogenesis and triggers histone hyperacetylation in zebrafish testes. Environ pollut. (2019) 248:368–79. doi: 10.1016/j.envpol.2019.01.127 30818116

[149] Lombó M Herráez MP. Paternal inheritance of bisphenol A cardiotoxic effects: the implications of sperm epigenome. Int J Mol Sci. (2021) 22:2125. doi: 10.3390/ijms22042125 33672782 PMC7924642

[150] Mo J Au DW Wan MT Shi J Zhang G Winkler C. Multigenerational impacts of benzo[a]pyrene on bone modeling and remodeling in medaka (Oryzias latipes). Environ Sci Technol. (2020) 54:12271–84. doi: 10.1021/acs.est.0c02416 32840350

[151] Saban JM Watson-Lazowski A Chapman MA Taylor G. The methylome is altered for plants in a high CO2 world: Insights into the response of a wild plant population to multigenerational exposure to elevated atmospheric [CO2. Glob Chang Biol. (2020) 26:6474–92. doi: 10.1111/gcb.15249 32902071

[152] Wu J Ren C Delfino RJ Chung J Wilhelm M Ritz B. Association between local traffic-generated air pollution and preeclampsia and preterm delivery in the south coast air basin of California. Environ Health Perspectives. (2009) 117:1773–9. doi: 10.1289/ehp.0800334 PMC2801174 20049131

[153] Ahn TG Kim YJ Lee G You YA Kim SM Chae R. Association between individual air pollution (PM10, PM2.5) exposure and adverse pregnancy outcomes in korea: A multicenter prospective cohort, air pollution on pregnancy outcome (APPO) study. J Korean Med Sci. (2024) 39:e131. doi: 10.3346/jkms.2024.39.e131 38599601 PMC11004777

[154] Twu O Dessí D Vu A Mercer F Stevens GC de Miguel N. Trichomonas vaginalis homolog of macrophage migration inhibitory factor induces prostate cell growth, invasiveness, and inflammatory responses. Proc Natl Acad Sci U S A. (2014) 111:8179–84. doi: 10.1073/pnas.1321884111 PMC4050605 24843155

[155] Onderdonk AB Delaney ML Fichorova RN. The human microbiome during bacterial vaginosis. Clin Microbiol Rev. (2016) 29:223–38. doi: 10.1128/CMR.00075-15 PMC4786887 26864580

[156] Aiyar A Quayle AJ Buckner LR Sherchand SP Chang TL Zea AH. Influence of the tryptophan-indole-IFNγ axis on human genital Chlamydia trachomatis infection: role of vaginal co-infections. Front Cell Infect Microbiol. (2014) 4:72. doi: 10.3389/fcimb.2014.00072 24918090 PMC4042155

[157] Sodhani P Gupta S Gupta R Mehrotra R. Bacterial vaginosis and cervical intraepithelial neoplasia: is there an association or is co-existence incidental? Asian Pac J Cancer Prev. (2017) 18:1289–92. doi: 10.22034/APJCP.2017.18.5.1289 PMC5555537 28610416

[158] Fellous A Wegner KM John U Mark FC Shama LNS. Windows of opportunity: Ocean warming shapes temperature-sensitive epigenetic reprogramming and gene expression across gametogenesis and embryogenesis in marine stickleback. Glob Chang Biol. (2022) 28:54–71. doi: 10.1111/gcb.15942 34669228

[159] Bertin G Averbeck D. Cadmium: cellular effects, modifications of biomolecules, modulation of DNA repair and genotoxic consequences (a review). Biochimie. (2006) 88:1549–59. doi: 10.1016/j.biochi.2006.10.001 17070979

[160] Carvan MJ 3rd Kalluvila TA Klingler RH Larson JK Pickens M Mora-Zamorano FX. Mercury-induced epigenetic transgenerational inheritance of abnormal neurobehavior is correlated with sperm epimutations in zebrafish. PloS One. (2017) 12:e0176155. doi: 10.1371/journal.pone.0176155 28464002 PMC5413066

[161] Bhan A Sarkar NN. Mercury in the environment: effect on health and reproduction. Rev Environ Health. (2005) 20:39–56. doi: 10.1515/reveh.2005.20.1.39 15835497

[162] Martos SN Tang WY Wang Z. Elusive inheritance: Transgenerational effects and epigenetic inheritance in human environmental disease. Prog Biophys Mol Biol. (2015) 118:44–54. doi: 10.1016/j.pbiomolbio.2015.02.011 25792089 PMC4784256

[163] Hou L Wang D Baccarelli A. Environmental chemicals and microRNAs. Mutat Res. (2011) 714:105–12. doi: 10.1016/j.mrfmmm.2011.05.004 PMC3739302 21609724

[164] Cao Y Calafat AM Doerge DR Umbach DM Bernbaum JC Twaddle NC. Isoflavones in urine, saliva, and blood of infants: Data from a pilot study on the estrogenic activity of soy formula. J Expo. Sci Environ Epidemiol. (2008) 19:223–34. doi: 10.1038/jes.2008.44 PMC2630504 18665197

[165] Yu L Rios E Castro L Liu J Yan Y Dixon D. Genistein: dual role in women's health. Nutrients. (2021) 13:3048. doi: 10.3390/nu13093048 34578926 PMC8472782

[166] Longnecker MP Klebanoff MA Zhou H Brock JW. Association between maternal serum concentration of the DDT metabolite DDE and preterm and small-for-gestational-age babies at birth. Lancet. (2001) 358:110–4. doi: 10.1016/S0140-6736(01)05329-6 11463412

[167] Gapp K von Ziegler L Tweedie-Cullen RY Mansuy IM. Early life epigenetic programming and transmission of stress-induced traits in mammals: how and when can environmental factors influence traits and their transgenerational inheritance? Bioessays. (2014) 36:491–502. doi: 10.1002/bies.201300116 24585414

[168] Marín-Palma D Tabares-Guevara JH Taborda N Rugeles MT Hernandez JC. Coarse particulate matter (PM10) induce an inflammatory response through the NLRP3 activation. J Inflammation (Lond). (2024) 21:15. doi: 10.1186/s12950-024-00388-9 PMC11064351 38698414

[169] Bollati V Marinelli B Apostoli P Bonzini M Nordio F Hoxha M. Exposure to metal-rich particulate matter modifies the expression of candidate microRNAs in peripheral blood leukocytes. Environ Health Perspect. (2010) 118:763–8. doi: 10.1289/ehp.0901300 PMC2898851 20061215

[170] Zhang TN Li D Wu QJ Xia J Wen R Chen XC. Exposure to nitrogen oxide in the first trimester and risk of cardiovascular-related malformations: A dose-response meta-analysis of observational studies. BioMed Res Int. (2018) 2018:1948407. doi: 10.1155/2018/1948407 29850486 PMC5914127

[171] Jarrett BY Vanden Brink H Brooks ED Hoeger KM Spandorfer SD Pierson RA. Impact of right-left differences in ovarian morphology on the ultrasound diagnosis of polycystic ovary syndrome. Fertil Steril. (2019) 112:939–46. doi: 10.1016/j.fertnstert.2019.06.016 PMC6858942 31395310

[172] Mimouni NEH Paiva I Barbotin AL Timzoura FE Plassard D Le Gras S. Polycystic ovary syndrome is transmitted via a transgenerational epigenetic process. Cell Metab. (2021) 33:513–530.e8. doi: 10.1016/j.cmet.2021.01.004 33539777 PMC7928942

[173] Olaniyi KS Areloegbe SE. Acetate circumvents impaired metabolic switch in skeletal muscle of letrozole-induced PCOS rat model by suppression of PDK4/NLRP3. Nutrition. (2023) 107:111914. doi: 10.1016/j.nut.2022.111914 36521396

[174] Geng X Zhao J Huang J Li S Chu W Wang WS. lnc-MAP3K13-7:1 inhibits ovarian GC proliferation in PCOS via DNMT1 downregulation-mediated CDKN1A promoter hypomethylation. Mol Ther. (2021) 29:1279–93. doi: 10.1016/j.ymthe.2020.11.018 PMC7934583 33212300

[175] Liu Y Zhang S Chen L Huang X Wang M Ponikwicka-Tyszko D. The molecular mechanism of miR-96-5p in the pathogenesis and treatment of polycystic ovary syndrome. Transl Res. (2023) 256:1–13. doi: 10.1016/j.trsl.2022.12.007 36586536

[176] Ling L Feng X Wei T Wang Y Wang Y Wang Z. Human amnion-derived mesenchymal stem cell (hAD-MSC) transplantation improves ovarian function in rats with premature ovarian insufficiency (POI) at least partly through a paracrine mechanism. Stem Cell Res Ther. (2019) 10:46. doi: 10.1186/s13287-019-1136-x 30683144 PMC6347748

[177] Huang B Ding C Zou Q Wang W Li H. Cyclophosphamide regulates N6-methyladenosine and m6A RNA enzyme levels in human granulosa cells and in ovaries of a premature ovarian aging mouse model. Front Endocrinol (Lausanne). (2019) 10:415. doi: 10.3389/fendo.2019.00415 31316467 PMC6610338

[178] Zhao BS Roundtree IA He C. Post-transcriptional gene regulation by mRNA modifications. Nat Rev Mol Cell Biol. (2017) 18:31–42. doi: 10.1038/nrm.2016.132 27808276 PMC5167638

[179] Mu H Zhang T Yang Y Zhang D Gao J Li J. METTL3-mediated mRNA N-methyladenosine is required for oocyte and follicle development in mice. Cell Death Dis. (2021) 12:989. doi: 10.1038/s41419-021-04272-9 34689175 PMC8542036

[180] Liu HB Muhammad T Guo Y Li MJ Sha QQ Zhang CX. RNA-binding protein IGF2BP2/IMP2 is a critical maternal activator in early zygotic genome activation. Advanced Sci (Weinheim Baden-Wurttemberg Germany). (2019) 6,15:1900295. doi: 10.1002/advs.201900295 PMC6685478 31406667

[181] Mei NH Guo SM Zhou Q Zhang YR Liu XZ Yin Y. H3K4 methylation promotes expression of mitochondrial dynamics regulators to ensure oocyte quality in mice. Adv Sci (Weinh). (2023) 10:e2204794. doi: 10.1002/advs.202204794 36815388 PMC10131798

[182] Wu SC Zhang Y. Minireview: role of protein methylation and demethylation in nuclear hormone signaling. Mol Endocrinol. (2009) 23:1323–34. doi: 10.1210/me.2009-0131 PMC2737564 19407220

[183] Zhang X Tanaka K Yan J Li J Peng D Jiang Y. Regulation of estrogen receptor α by histone methyltransferase SMYD2-mediated protein methylation. Proc Natl Acad Sci U S A. (2013) 110:17284–9. doi: 10.1073/pnas.1307959110 PMC3808627 24101509

[184] Wang L Ozark PA Smith ER Zhao Z Marshall SA Rendleman EJ. TET2 coactivates gene expression through demethylation of enhancers. Sci Adv. (2018) 4:eaau6986. doi: 10.1126/sciadv.aau6986 30417100 PMC6221537

[185] Bulun SE Yildiz S Adli M Chakravarti D Parker JB Milad M. Endometriosis and adenomyosis: shared pathophysiology. Fertil Steril. (2023) 119:746–50. doi: 10.1016/j.fertnstert.2023.03.006 36925057

[186] Cui J Wu F Yang X Liu S Han S Chen B. Effects of ammonia on hypothalamic-pituitary-ovarian axis in female rabbits. Ecotoxicol Environ Saf. (2021) 227:112922. doi: 10.1016/j.ecoenv.2021.112922 34700170

[187] Steegers-Theunissen RPM Wiegel RE Jansen PW Laven JSE Sinclair KD. Polycystic ovary syndrome: A brain disorder characterized by eating problems originating during puberty and adolescence. Int J Mol Sci. (2020) 21:8211. doi: 10.3390/ijms21218211 33153014 PMC7663730

[188] Wang A Wan Y Mahai G Qian X Li Y Xu S. Association of prenatal exposure to organophosphate, pyrethroid, and neonicotinoid insecticides with child neurodevelopment at 2 years of age: A prospective cohort study. Environ Health Perspect. (2023) 131:107011. doi: 10.1289/EHP12097 37856202 PMC10586492

[189] Cheong AW Pang RT Liu WM Kottawatta KS Lee KF Yeung WS. MicroRNA Let-7a and dicer are important in the activation and implantation of delayed implanting mouse embryos. Hum Reprod. (2014) 29:750–62. doi: 10.1093/humrep/det462 24419497

[190] Andersson KL Bussani C Fambrini M Polverino V Taddei GL Gemzell-Danielsson K. DNA methylation of HOXA10 in eutopic and ectopic endometrium. Hum Reprod. (2014) 29:1906–11. doi: 10.1093/humrep/deu161 24963168

[191] Taylor HS Arici A Olive D Igarashi P. HOXA10 is expressed in response to sex steroids at the time of implantation in the human endometrium. J Clin Invest. (1998) 101:1379–84. doi: 10.1172/JCI1057 PMC508715 9525980

[192] Cao C Zhou Y Zhang Y Ma Y Du S Fan L. GCN5 participates in KLF4-VEGFA feedback to promote endometrial angiogenesis. iScience. (2022) 25:104509. doi: 10.1016/j.isci.2022.104509 35733790 PMC9207667

[193] Revel A Achache H Stevens J Smith Y Reich R. MicroRNAs are associated with human embryo implantation defects. Hum Reprod. (2011) 26:2830–40. doi: 10.1093/humrep/der255 21849299

[194] Li Y Liu H Ye S Zhang B Li X Yuan J. The effects of coagulation factors on the risk of endometriosis: a Mendelian randomization study. BMC Med. (2023) 21:195. doi: 10.1186/s12916-023-02881-z 37226166 PMC10210381

[195] Beddows I Fan H Heinze K Johnson BK Leonova A Senz J. Cell state of origin impacts development of distinct endometriosis-related ovarian carcinoma histotypes. Cancer Res. (2024) 84:26–38. doi: 10.1158/0008-5472.CAN-23-1362 37874327 PMC10758692

[196] Mortlock S Houshdaran S Kosti I Rahmioglu N Nezhat C Vitonis AF. Global endometrial DNA methylation analysis reveals insights into mQTL regulation and associated endometriosis disease risk and endometrial function. Commun Biol. (2023) 6:780. doi: 10.1038/s42003-023-05070-z 37587191 PMC10432557

[197] Peinado FM Olivas-Martínez A Iribarne-Durán LM Ubiña A León J Vela-Soria F. Cell cycle, apoptosis, cell differentiation, and lipid metabolism gene expression in endometriotic tissue and exposure to parabens and benzophenones. Sci Total Environ. (2023) 879:163014. doi: 10.1016/j.scitotenv.2023.163014 37003176

[198] Kim TH Yoo JY Choi KC Shin JH Leach RE Fazleabas AT. Loss of HDAC3 results in nonreceptive endometrium and female infertility. Sci Transl Med. (2019) 11:eaaf7533. doi: 10.1126/scitranslmed.aaf7533 30626716 PMC6650287

[199] Moustafa S Burn M Mamillapalli R Nematian S Flores V Taylor HS. Accurate diagnosis of endometriosis using serum microRNAs. Am J obstetrics gynecology. (2020) 223:557.e1–557.e11. doi: 10.1016/j.ajog.2020.02.050 32165186

[200] Ma'ayeh M Costantine MM. Prevention of preeclampsia. Semin Fetal Neonatal Med. (2020) 25:101123. doi: 10.1016/j.siny.2020.101123 32513597 PMC8236336

[201] Caniggia I Lye SJ Cross JC. Activin is a local regulator of human cytotrophoblast cell differentiation. Endocrinology. (1997) 138:3976–86. doi: 10.1210/endo.138.9.5403 9275089

[202] Moses EK Lade JA Guo G Wilton AN Grehan M Freed K. A genome scan in families from Australia and New Zealand confirms the presence of a maternal susceptibility locus for pre-eclampsia, on chromosome 2. Am J Hum Genet. (2000) 67:1581–5. doi: 10.1086/316888 PMC1287935 11035632

[203] Jones RL Salamonsen LA Findlay JK. Activin A promotes human endometrial stromal cell decidualization in vitro. J Clin Endocrinol Metab. (2002) 87:4001–4. doi: 10.1210/jcem.87.8.8880 12161551

[204] Pados G Gordts S Sorrentino F Nisolle M Nappi L Daniilidis A. Adenomyosis and infertility: A literature review. Medicina. (2023) 59:1551. doi: 10.3390/medicina59091551 37763670 PMC10534714

[205] Zhai J Vannuccini S Petraglia F Giudice LC. Adenomyosis: mechanisms and pathogenesis. Semin Reprod Med. (2020) 38:129–43. doi: 10.1055/s-0040-1716687 PMC7932680 33032339

[206] Huang E Chen L. RNA N6-methyladenosine modification in female reproductive biology and pathophysiology. Cell Commun Signal. (2023) 21:53. doi: 10.1186/s12964-023-01078-4 36894952 PMC9996912

[207] Wu Y Yang R Lan J Wu Y Huang J Fan Q. Iron overload modulates follicular microenvironment via ROS/HIF-1α/FSHR signaling. Free Radic Biol Med. (2023) 196:37–52. doi: 10.1016/j.freeradbiomed.2022.12.105 36638901

[208] Liu B Zhai J Wang W Liu T Liu C Zhu X. Identification of tumor microenvironment and DNA methylation-related prognostic signature for predicting clinical outcomes and therapeutic responses in cervical cancer. Front Mol Biosci. (2022) 9:872932. doi: 10.3389/fmolb.2022.872932 35517856 PMC9061945

[209] Navarro A Bariani MV Yang Q Al-Hendy A. Understanding the impact of uterine fibroids on human endometrium function. Front Cell Dev Biol. (2021) 9:633180. doi: 10.3389/fcell.2021.633180 34113609 PMC8186666

[210] Yang Q Mas A Diamond MP Al-Hendy A. The mechanism and function of epigenetics in uterine leiomyoma development. Reprod Sci. (2016) 23:163–75. doi: 10.1177/1933719115584449 PMC5933172 25922306

[211] Carbajo-García MC De Miguel-Gómez L Juárez-Barber E Trelis A Monleón J Pellicer A. Deciphering the role of histone modifications in uterine leiomyoma:acetylation of h3k27 regulates the expression of genes involved in proliferation,cell signaling,cell transport,angiogenesis and extracellular matrix formation. Biomedicines. (2022) 10(6):1279. doi: 10.3390/biomedicines10061279 35740301 PMC9219820

[212] Włodarczyk M Nowicka G Ciebiera M Ali M Yang Q Al-Hendy A. Epigenetic regulation in uterine fibroids—The role of ten-eleven translocation enzymes and their potential therapeutic application. Int J Mol Sci. (2022) 23:2720. doi: 10.3390/ijms23052720 35269864 PMC8910916

[213] Zhou W Wang G Li B Qu J Zhang Y. LncRNA APTR promotes uterine leiomyoma cell proliferation by targeting erα to activate the Wnt/β-catenin pathway. Front Oncol. (2021) 11:536346. doi: 10.3389/fonc.2021.536346 33777725 PMC7989393

[214] Zhao L Liang X Wang L Zhang X. The role of miRNA in ovarian cancer: an overview. Reprod Sci. (2022) 29:2760–7. doi: 10.1007/s43032-021-00717-w PMC9537199 34973152

[215] Di Leva G Croce CM. Roles of small RNAs in tumor formation. Trends Mol Med. (2010) 16:257–67. doi: 10.1016/j.molmed.2010.04.001 PMC2885513 20493775

[216] Xiang G Cheng Y. MiR-126-3p inhibits ovarian cancer proliferation and invasion via targeting PLXNB2. Reprod Biol. (2018) 18:218–24. doi: 10.1016/j.repbio.2018.07.005 30054097

[217] Vernon M Lambert B Meryet-Figuière M Brotin E Weiswald LB Paysant H. Functional miRNA Screening Identifies Wide-ranging Antitumor Properties of miR-3622b-5p and Reveals a New Therapeutic Combination Strategy in Ovarian Tumor Organoids. Mol Cancer Ther. (2020) 19:1506–19. doi: 10.1158/1535-7163.MCT-19-0510 32371581

[218] Ibanez de Caceres I Battagli C Esteller M Herman JG Dulaimi E Edelson MI. Tumor cell-specific BRCA1 and RASSF1A hypermethylation in serum, plasma, and peritoneal fluid from ovarian cancer patients. Cancer Res. (2004) 64:6476–81. doi: 10.1158/0008-5472.CAN-04-1529 15374957

[219] SK S Swamy SN Premalatha CS Pallavi VR Gawari R. Aberrant promoter hypermethylation of RASSF1a and BRCA1 in circulating cell-free tumor DNA serves as a biomarker of ovarian carcinoma. Asian Pac J Cancer Prev. (2019) 20:3001–5. doi: 10.31557/APJCP.2019.20.10.3001 PMC6982682 31653147

[220] Sen P Ganguly P Ganguly N. Modulation of DNA methylation by human papillomavirus E6 and E7 oncoproteins in cervical cancer. Oncol Lett. (2018) 15:11–22. doi: 10.3892/ol.2017.7292 29285184 PMC5738689

[221] Chung SH Franceschi S Lambert PF. Estrogen and ERalpha: culprits in cervical cancer? Trends Endocrinol Metab. (2010) 21:504–11. doi: 10.1016/j.tem.2010.03.005 PMC2914219 20456973

[222] Ma X Liu J Wang H Jiang Y Wan Y Xia Y. Identification of crucial aberrantly methylated and differentially expressed genes related to cervical cancer using an integrated bioinformatics analysis. Biosci Rep. (2020) 40:BSR20194365. doi: 10.1042/BSR20194365 32368784 PMC7218222

[223] Johansson C Jamal Fattah T Yu H Nygren J Mossberg AK Schwartz S. Acetylation of intragenic histones on HPV16 correlates with enhanced HPV16 gene expression. Virology. (2015) 482:244–59. doi: 10.1016/j.virol.2015.02.053 25900886

[224] Du Y Wei N Ma R Jiang SH Song D. Long noncoding RNA MIR210HG is induced by hypoxia-inducible factor 1α and promotes cervical cancer progression. Am J Cancer Res. (2022) 12:2783–97. doi: 10.21203/rs.3.rs-1207674/v1 PMC9251695 35812055

[225] Guha P Sen K Chowdhury P Mukherjee D. Estrogen receptors as potential therapeutic target in endometrial cancer. J Recept Signal Transduct Res. (2023) 43:19–26. doi: 10.1080/10799893.2023.2187643 36883690

[226] Ghabreau L Roux JP Niveleau A Fontanière B Mahe C Mokni M. Correlation between the DNA global methylation status and progesterone receptor expression in normal endometrium, endometrioid adenocarcinoma and precursors. Virchows Arch. (2004) 445:129–34. doi: 10.1007/s00428-004-1059-4 15221375

[227] Caplakova V Babusikova E Blahovcova E Balharek T Zelieskova M Hatok J. DNA methylation machinery in the endometrium and endometrial cancer. Anticancer Res. (2016) 36:4407–20. doi: 10.21873/anticanres.10984 27630276

[228] Kumar S Sharma A Kshetrimayum C. Environmental & occupational exposure & female reproductive dysfunction. Indian J Med Res. (2019) 150:532–45. doi: 10.4103/ijmr.IJMR_1652_17 PMC7038808 32048617

[229] Petrelli G Figà-Talamanca I Tropeano R Tangucci M Cini C Aquilani S. Reproductive male-mediated risk: spontaneous abortion among wives of pesticide applicators. Eur J Epidemiol. (2000) 16:391–3. doi: 10.1023/a:1007630610911 10959949

[230] Frazier LM. Reproductive disorders associated with pesticide exposure. J Agromedicine. (2007) 12:27–37. doi: 10.1300/J096v12n01_04 18032334

[231] Rogan WJ Chen A. Health risks and benefits of bis(4-chlorophenyl)-1,1,1-trichloroethane (DDT). Lancet. (2005) 366:763–73. doi: 10.1016/S0140-6736(05)67182-6 16125595

[232] Salazar-García F Gallardo-Díaz E Cerón-Mireles P Loomis D Borja-Aburto VH. Reproductive effects of occupational DDT exposure among male malaria control workers. Environ Health Perspect. (2004) 112:542–7. doi: 10.1289/ehp.112-1241918 PMC1241918 15064158

[233] Reshi MS Mustafa RA Javaid D Haque S. Pesticide Toxicity Associated with Infertility. Adv Exp Med Biol. (2022) 1319:59–69. doi: 10.1007/978-3-031-12966-7_4 36472816

[234] Hall MS Talge NM Upson K. Urinary cadmium and endometriosis prevalence in a US nationally representative sample: results from NHANES 1999-2006. Hum Reprod. (2023) 38:1835–42. doi: 10.1093/humrep/dead117 PMC10477936 37487110

[235] El Fouikar S Duranthon V Helies V Jammes H Couturier-Tarrade A Gayrard V. Multigenerational effects of a complex human-relevant exposure during folliculogenesis and preimplantation embryo development: the FEDEXPO study. Toxics. (2023) 11:425. doi: 10.3390/toxics11050425 37235240 PMC10222629

[236] Eckersley-Maslin MA Alda-Catalinas C Reik W. Dynamics of the epigenetic landscape during the maternal-to-zygotic transition. Nat Rev Mol Cell Biol. (2018) 19:436–50. doi: 10.1038/s41580-018-0008-z 29686419

[237] Jin Z Sheng J Hu Y Zhang Y Wang X Huang Y. Shining a spotlight on m6A and the vital role of RNA modification in endometrial cancer: a review. Front Genet. (2023) 14:1247309. doi: 10.3389/fgene.2023.1247309 37886684 PMC10598767

[238] Raja MHR Farooqui N Zuberi N Ashraf M Azhar A Baig R. Endometriosis, infertility and MicroRNA's: A review. J Gynecol Obstet Hum Reprod. (2021) 50:102157. doi: 10.1016/j.jogoh.2021.102157 33957270

[239] Nandi S Tripathi SK Singh PK Gupta PSP Mondal S. Global DNA methylation, DNA methyltransferase and stress-related gene expression in ovine oocytes and embryos after exposure to metabolic stressors. Reprod Domest Anim. (2023) 58:717–25. doi: 10.1111/rda.14341 36920043

[240] Ibrahim Y Hotaling J. Sperm epigenetics and its impact on male fertility, pregnancy loss, and somatic health of future offsprings. Semin Reprod Med. (2018) 36:233–9. doi: 10.1055/s-0038-1677047 30866010

[241] Gaspari L Paris F Soyer-Gobillard MO Kalfa N Sultan C Hamamah S. Perturbateurs endocriniens environnementaux et fertilité [Environmental endocrine disruptors and fertility. Gynecol Obstet Fertil Senol. (2022) 50:402–8. doi: 10.1016/j.gofs.2021.09.009 34560302

[242] Karwacka A Zamkowska D Radwan M Jurewicz J. Exposure to modern, widespread environmental endocrine disrupting chemicals and their effect on the reproductive potential of women: an overview of current epidemiological evidence. Hum Fertil (Camb). (2019) 22:2–25. doi: 10.1080/14647273.2017.1358828 28758506

[243] Gaskins AJ Mínguez-Alarcón L Williams PL Chavarro JE Schwartz JD Kloog I. Ambient air pollution and risk of pregnancy loss among women undergoing assisted reproduction. Environ Res. (2020) 191:110201. doi: 10.1016/j.envres.2020.110201 32937174 PMC7658021

[244] Hilbert SM Gunderson S. Complications of assisted reproductive technology. Emerg Med Clin North Am. (2019) 37:239–49. doi: 10.1016/j.emc.2019.01.005 30940369

[245] Lucas E. Epigenetic effects on the embryo as a result of periconceptional environment and assisted reproduction technology. Reprod BioMed Online. (2013) 27:477–85. doi: 10.1016/j.rbmo.2013.06.003 23933034

[246] Alves da Silva AF MaChado FB Pavarino ÉC Biselli-Périco JM Zampieri BL da Silva Francisco JR. Trisomy 21 alters DNA methylation in parent-of-origin-dependent and -independent manners. PloS One. (2016) 11:e0154108. doi: 10.1371/journal.pone.0154108 27100087 PMC4839675

[247] Sui X Hu Y Ren C Cao Q Zhou S Cao Y. METTL3-mediated m6A is required for murine oocyte maturation and maternal-to-zygotic transition. Cell Cycle. (2020) 19:391–404. doi: 10.1080/15384101.2019.1711324 31916488 PMC7100890

[248] Xia H Zhong C Wu X Chen J Tao B Xia X. Mettl3 mutation disrupts gamete maturation and reduces fertility in zebrafish. Genetics. (2018) 208:729–43. doi: 10.1534/genetics.117.300574 PMC5788534 29196300

[249] Niehrs C Calkhoven CF. Emerging role of C/EBPβ and epigenetic DNA methylation in ageing. Trends Genet. (2020) 36:71–80. doi: 10.1016/j.tig.2019.11.005 31822355

[250] Andraos S de Seymour JV O'Sullivan JM Kussmann M. The impact of nutritional interventions in pregnant women on DNA methylation patterns of the offspring: A systematic review. Mol Nutr Food Res. (2018) 62:e1800034. doi: 10.1002/mnfr.201800034 30035846

[251] Goodrich JM Reddy P Naidoo RN Asharam K Batterman S Dolinoy DC. Prenatal exposures and DNA methylation in newborns: a pilot study in Durban, South Africa. Environ Sci Process Impacts. (2016) 18:908–17. doi: 10.1039/c6em00074f PMC4945397 27359112

[252] Delhaes F Giza SA Koreman T Eastabrook G McKenzie CA Bedell S. Altered maternal and placental lipid metabolism and fetal fat development in obesity: Current knowledge and advances in non-invasive assessment. Placenta. (2018) :69:118–124. doi: 10.1016/j.placenta.2018.05.011 29907450

[253] Bowman CE Arany Z Wolfgang MJ. Regulation of maternal-fetal metabolic communication. Cell Mol Life Sci. (2021) 78:1455–86. doi: 10.1007/s00018-020-03674-w PMC7904600 33084944

[254] Kalish JM Jiang C Bartolomei MS. Epigenetics and imprinting in human disease. Int J Dev Biol. (2014) 58:291–8. doi: 10.1387/ijdb.140077mb 25023695

[255] Joó JG Karabélyos C Héjja H Kornya L Rigó J Jr. Epigenetikai mechanizmusok élettani és kóros terhességben [Epigenetic mechanisms in physiologic and pathologic pregnancies. Orv Hetil. (2014) 155:566–74. doi: 10.1556/OH.2014.29861 24704768

[256] Ventura-Juncá P Irarrázaval I Rolle AJ Gutiérrez JI Moreno RD Santos MJ. *In vitro* fertilization (IVF) in mammals: epigenetic and developmental alterations. Sci bioethical implications IVF humans. Biol Res. (2015) 48:68. doi: 10.1186/s40659-015-0059-y PMC4684609 26683055

[257] Hansen PJ Dobbs KB Denicol AC Siqueira LGB. Sex and the preimplantation embryo: implications of sexual dimorphism in the preimplantation period for maternal programming of embryonic development. Cell Tissue Res. (2016) 363:237–47. doi: 10.1007/s00441-015-2287-4 PMC4703572 26391275

[258] Johnson JP Beischel L Schwanke C Styren K Crunk A Schoof J. Overrepresentation of pregnancies conceived by artificial reproductive technology in prenatally identified fetuses with Beckwith-Wiedemann syndrome. J Assist Reprod Genet. (2018) 35:985–92. doi: 10.1007/s10815-018-1228-z PMC6030004 29936652

[259] Li Piani L Reschini M Somigliana E Ferrari S Busnelli A Viganò P. telomere length and DNA methylation as predictors of live birth in *in vitro* fertilization cycles. PloS One. (2022) 17:e0261591. doi: 10.1371/journal.pone.0261591 35073322 PMC8786209

[260] Ding T Yan W Zhou T Shen W Wang T Li M. Endocrine disrupting chemicals impact on ovarian aging: Evidence from epidemiological and experimental evidence. Environ pollut. (2022) 305:119269. doi: 10.1016/j.envpol.2022.119269 35405219

[261] Muraki Y Banno K Yanokura M Kobayashi Y Kawaguchi M Nomura H. Relationship of aberrant DNA hypermethylation of CHFR with sensitivity to taxanes in endometrial cancer. Oncol Rep. (2009) 22(5):967–72. doi: 10.3892/or_00000523 17143476

[262] Hassanzadeh M Mahernia S Caprini G Fossati G Adib M Moakedi F. Epigenetic-based cancer therapeutics: new potential HDAC8 inhibitors. J Biomol Struct Dyn. (2022) 40:297–311. doi: 10.1080/07391102.2020.1813203 32886033

[263] Shawky H Tawfik H Hewidy M. Weekly dose-dense paclitaxel and carboplatin in recurrent ovarian carcinoma: a phase II trial. J Egypt Natl Canc Inst. (2014) 26:139–45. doi: 10.1016/j.jnci.2014.05.001 25150129

[264] Migliore L Coppedè F. Gene-environment interactions in Alzheimer disease: the emerging role of epigenetics. Nat Rev Neurol. (2022) 18:643–60. doi: 10.1038/s41582-022-00714-w 36180553

[265] Liu X Guo S-W. Aberrant immunoreactivity of deoxyribonucleic acid methyltransferases in adenomyosis. Gynecol Obstet Invest. (2012) 74:100–8. doi: 10.1159/000337718 22572543

[266] Kim M Kang D Kwon MY Lee HJ Kim MJ. MicroRNAs as potential indicators of the development and progression of uterine leiomyoma. PloS One. (2022) 17:e0268793.microRNA. doi: 10.1371/journal.pone.0268793 35639702 PMC9154092

[267] Gao Y Zhou N Liu J. Ovarian cancer diagnosis and prognosis based on cell-free DNA methylation. Cancer Control. (2024) 31:10732748241255548. doi: 10.1177/10732748241255548 38764160 PMC11104031

[268] Sun L Mu Y Xu L Han X Gu W Zhang M. Transgenerational inheritance of wing development defects in Drosophila melanogaster induced by cadmium. Ecotoxicol Environ Saf. (2023) 250:114486. doi: 10.1016/j.ecoenv.2022.114486 36587412

